# Novel caffeoylquinic acid derivatives from *Lonicera japonica* Thunb. flower buds exert pronounced anti-HBV activities†

**DOI:** 10.1039/c8ra07549b

**Published:** 2018-10-15

**Authors:** Lanlan Ge, Haoqiang Wan, Shuming Tang, Haixia Chen, Jiemei Li, Keda Zhang, Boping Zhou, Jia Fei, Shiping Wu, Xiaobin Zeng

**Affiliations:** Center Lab of Longhua Branch, Shenzhen People's Hospital, 2nd Clinical Medical College of Jinan University Shenzhen 518120 Guangdong Province China zengxiaobin1983@163.com +86-755-28100877 +86-755-27745118; Department of Infectious Disease, Shenzhen People's Hospital, 2nd Clinical Medical College of Jinan University Shenzhen 518120 Guangdong Province China wupoem@126.com; Integrated Chinese and Western Medicine Postdoctoral Research Station, Jinan University Guangzhou 510632 Guangdong Province China; Department of Pathology (Longhua Branch), Shenzhen People's Hospital, 2nd Clinical Medical College of Jinan University Shenzhen 518120 Guangdong Province China; Laboratory Department of Longhua Branch, Shenzhen People's Hospital, 2nd Clinical Medical College of Jinan University Shenzhen 518120 Guangdong Province China

## Abstract

*Lonicera japonica* Thunb., possesses antiviral and hepatoprotective activities, and is widely used as a health food and in cosmetics. However, its major constituents, caffeoylquinic acid derivatives, and their anti-HBV activity were lacking systematic research. In this study, four novel caffeoylquinic acids, five simple caffeic acids and fourteen known caffeoylquinic acids are isolated and identified from *L. japonica*. Most caffeoylquinic acids inhibited HBsAg and HBeAg secretion, and HBV DNA replication. In particular, 100 μg ml^−1^ monocaffeoylquinic acid 9 inhibits HBsAg and HBeAg secretion, and HBV DNA replication by 83.82, 70.76 and 39.36% compared to the control. Unfortunately, 50 μg ml^−1^ tricaffeoylquinic acid 23 promotes HBsAg and HBeAg secretion, and HBV DNA replication by 172.39, 9.92 and 55.40%. Finally, structure–activity relationships reveal that caffeoylquinic acids containing a caffeoyl group have better inhibitory activities. The results indicate that caffeoylquinic acids from *L. japonica* could serve as anti-HBV agents for functional food or medicinal use.

## Abstract 2

*Lonicera japonica* Thunb., possesses antiviral and hepatoprotective activities, and is widely used as a health food and in cosmetics.

## Graphical abstract

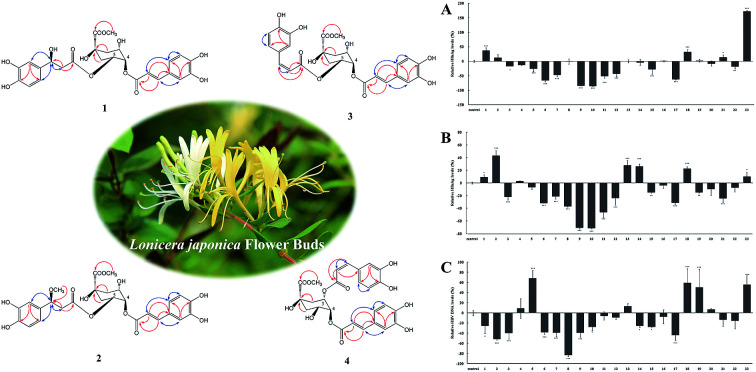

## Introduction

The hepatitis B virus (HBV) is a major global health problem, due to its worldwide distribution and potential adverse consequences. Current therapeutic strategies for HBV, however, are far from satisfactory.^1^ Traditional Chinese medicines, with multiple components and diverse activities, are important sources for novel anti-HBV drug discoveries. Caffeoylquinic acids, which exist widely in the plant kingdom, especially in Asteraceae,^2^ Umbelliferae,^3^ and Caprifoliaceae,^4^ are a type of polyphenol compound formed by esterification and condensation of a molecule of quinic acid and a multimolecule caffeic acid. Caffeoylquinic acids and their derivatives are reported to possess anti-HBV activities.^5,6^

*Lonicera japonica* Thunb. (Caprifoliaceae) is native to East Asia. In China, *L. japonica* is widely cultivated in Shandong and Henan Provinces. Jin Yin Hua, also known as Ren Dong, the flower buds of *L. japonica*, are famous in traditional Chinese medicine.^7^ During the recent decades, they have been used for treating influenza, cold, fever, and infections.^8^ Moreover, they have also been used in indigenous beverages, such as tea, in Korea and China for many years.^9^ Previous phytochemical investigations have been reported for different types of chemical constituents, including flavonoids, caffeoylquinic acids, iridoids, saponins, and other compounds.^9–12^ Furthermore, caffeoylquinic acids are major constituents of *L. japonica*.^13,14^ However, the caffeoylquinic acid derivatives form *L. japonica* and their anti-HBV activities are lacking in systematic research.

**Fig. 1:**
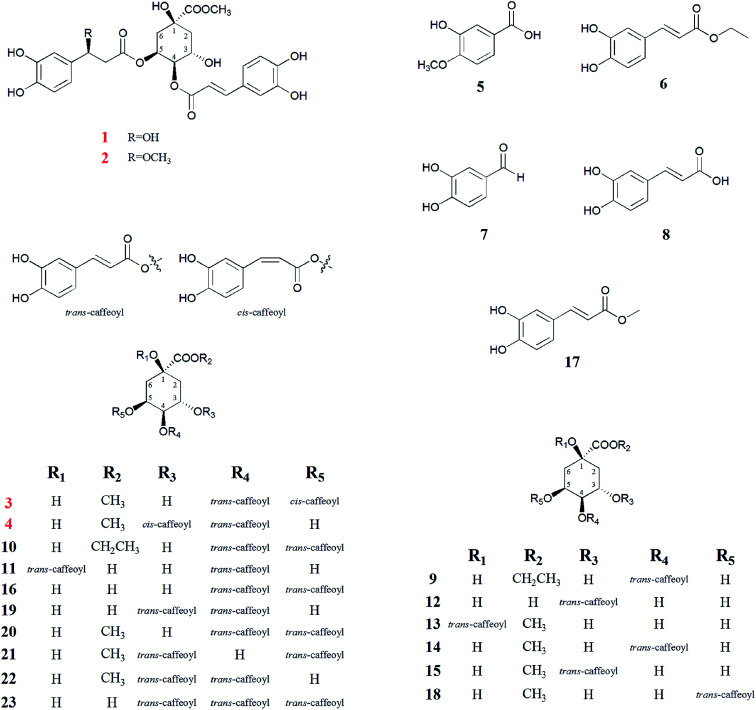
Chemical structure of all caffeoylquinic acid derivatives (1–23) in *Lonicera japonica* flower buds.

To clarify the anti-HBV activity of caffeoylquinic acids from the flower buds of *L. japonica* Thunb., as well as four new caffeoylquinic acids (1–4), together with five simple caffeic acids (5–8, 17), and fourteen known caffeoylquinic acids (9–16, 18–23) are systematically isolated and identified (Fig. 1). More importantly, most caffeoylquinic acids exhibit significantly anti-HBV activity, especially caffeoylquinic acids (6, 7, 9–12, 17). Furthermore, compound 23 (tricaffeoylquinic acid) appear to promote HBsAg and HBeAg secretion, and HBV DNA replication. Finally, the detailed structure–activity relationships of caffeoylquinic acids will be discussed in this research.

## Experimental

### General experimental procedures

Nuclear magnetic resonance (NMR) spectra were recorded on a Bruker DPX-400 spectrometer using standard Bruker pulse programs (Bruker, Karlsruhe, Germany). Chemical shifts are shown as *δ*-values with reference to tetramethylsilane (TMS) as an internal standard. Electrospray ionization mass spectrometry (ESI-MS) data was obtained on a Bruker Esquire LC 200-Ion trap mass spectrometer (Bruker, Karlsruhe, Germany), and high-resolution ESI-MS (HR-ESIMS) were measured on a Bruker microTOF-QII mass spectrometer (Bruker, Karlsruhe, Germany). Optical rotations were measured using a JASCO P-1020 automatic digital polarimeter (Jasco International, Tokyo, Japan). Sephadex LH-20 (GE, America), silica gel (Qingdao Ocean Chemical Co., Ltd, Qingdao, China), and ODS (40–63 μm, Merck, Darmstadt, Germany) were used for column chromatography. Thin-layer chromatography (TLC) was carried out on silica gel 60 F_254_ (Qingdao Ocean Chemical Co., Ltd, Qingdao, China), and spots were visualized by spraying the plates with 10% H_2_SO_4_ in EtOH and heating them at 105 °C. Preparative high-performance liquid chromatography (HPLC) was carried out on a EasyChrom 3.2.8.0 system (Guangzhou Ruibai Instrument Technology Co. Ltd., Guangzhou, China) and an octadecyl silica (ODS) column (Cosmosil 5C_18_-MS-II, 20 × 250 mm, Nacalai Tesque, Kyoto, Japan). Diagnostic kits for HBsAg and HBeAg were purchased from Abbott Trading Shanghai Co. Ltd. (Shanghai, China), and a diagnostic kit for HBV DNA was purchased from Hunan Shengxiang Jiancheng Biotechnology Co. Ltd. (Hunan, China). HPLC-grade methanol was purchased from Sigma-Aldrich Co. (St. Louis, USA). All other analytical chemicals were obtained from Shanghai Chemical Reagents Co., Ltd (Shanghai, China).

### Plant materials

The flower buds of *L. japonica* were purchased in September 2017 from Fukangwanjia Pharmaceuticals, Haozhou, Anhui Province, People's Republic of China. The plants were identified by Dr XB Zeng of Shenzhen People's Hospital and a voucher specimen (no. 20170930) was deposited at Center Lab of Longhua Branch, Shenzhen People's Hospital, Second Clinical Medical College of Jinan University, Shenzhen, China.

### Extraction and isolation

The air-dried and powdered *L. japonic*a flower buds (6.5 kg) were extracted three times with 75% ethanol (EtOH, 3 × 50 L) under room temperature and concentrated by evaporation *in vacuo*. Then the EtOH extract (1.5 kg) was suspended in distilled water and successively partitioned three times with cyclohexane, ethyl acetate (EtOAc), and *n*-butyl alcohol, respectively. The EtOAc fraction (83 g) was separated by silica gel column chromatography (5 × 45 cm, 100–200 mesh, 1800 g) eluted with a gradient of dichloromethane-methanol (CH_2_Cl_2_–MeOH, 100 : 1 → 1 : 0, v/v) to afford 20 fractions (fractions 1–20) by TLC plate analysis.

Fr. 4 (yellow colloidal, 2.9 g) was subjected to Sephadex LH-20 column chromatography (2.3 × 75 cm) isocratic eluted with CH_2_Cl_2_–MeOH (80 : 20, v/v) to afford 6 subfractions (Fr. 4–1 to Fr. 4–6). Next, compound 5 (50.0 mg) was obtained through recrystallization in methanol from Fr. 4–6.

Fr. 6 (yellow colloidal, 2.0 g) was separated by Sephadex LH-20 column chromatography (2.3 × 75 cm, CH_2_Cl_2_–MeOH, 80 : 20, v/v) to yield 12 subfractions (Fr. 6–1 to Fr. 6–12). Fr. 6–9 was subjected to preparative HPLC (Cosmosil 5C_18_-MS-II, 5 μm, 20 × 250 mm, flow rate: 8 ml min^−1^, wave length: 330 nm, MeOH : H_2_O, 57 : 43, v/v) yielding compound 6 (10.0 mg, *t*_R_: 19.3 min). Fr. 6–11 was also purified by preparative HPLC. HPLC (Cosmosil 5C_18_-MS-II, 5 μm, 20 × 250 mm, flow rate: 8 ml min^−1^, wave length: 330 nm, MeOH : H_2_O, 58 : 42, v/v) to afford compound 7 (3.0 mg, *t*_R_: 7.7 min).

Fr. 10 (yellow oil, 3.0 g) was submitted to Sephadex LH-20 column chromatography (2.3 × 75 cm) isocratic eluted with CH_2_Cl_2_–MeOH (80 : 20, v/v) to afford 4 subfractions (Fr. 10–1 to Fr. 10–4). Fr. 10–3 was applied to preparative HPLC (Cosmosil 5C_18_-MS-II, 5 μm, 20 × 250 mm, flow rate: 8 ml min^−1^, wave length: 330 nm, MeOH : H_2_O, 57 : 43, v/v) and obtain compound 8 (60 mg, *t*_R_: 7.7 min).

Fr. 13 (yellow solid, 5.5 g) was chromatographed on Sephadex LH-20 column (2.3 × 75 cm, CH_2_Cl_2_–MeOH, 80 : 20, v/v) to afford subfractions Fr. 13–1 to 13–8 *via* TLC, and Fr. 13–4 was subjected to preparative HPLC (Cosmosil 5C_18_-MS-II, 5 μm, 20 × 250 mm, flow rate: 8 ml min^−1^, wave length: 330 nm, MeOH : H_2_O, 55 : 45, v/v) yielding compound 9 (13.7 mg, *t*_R_: 9.2 min), compound 10 (18.7 mg, *t*_R_: 12.1 min) and compound 11 (2.1 mg, *t*_R_: 19.2 min).

**Fig. 2:**
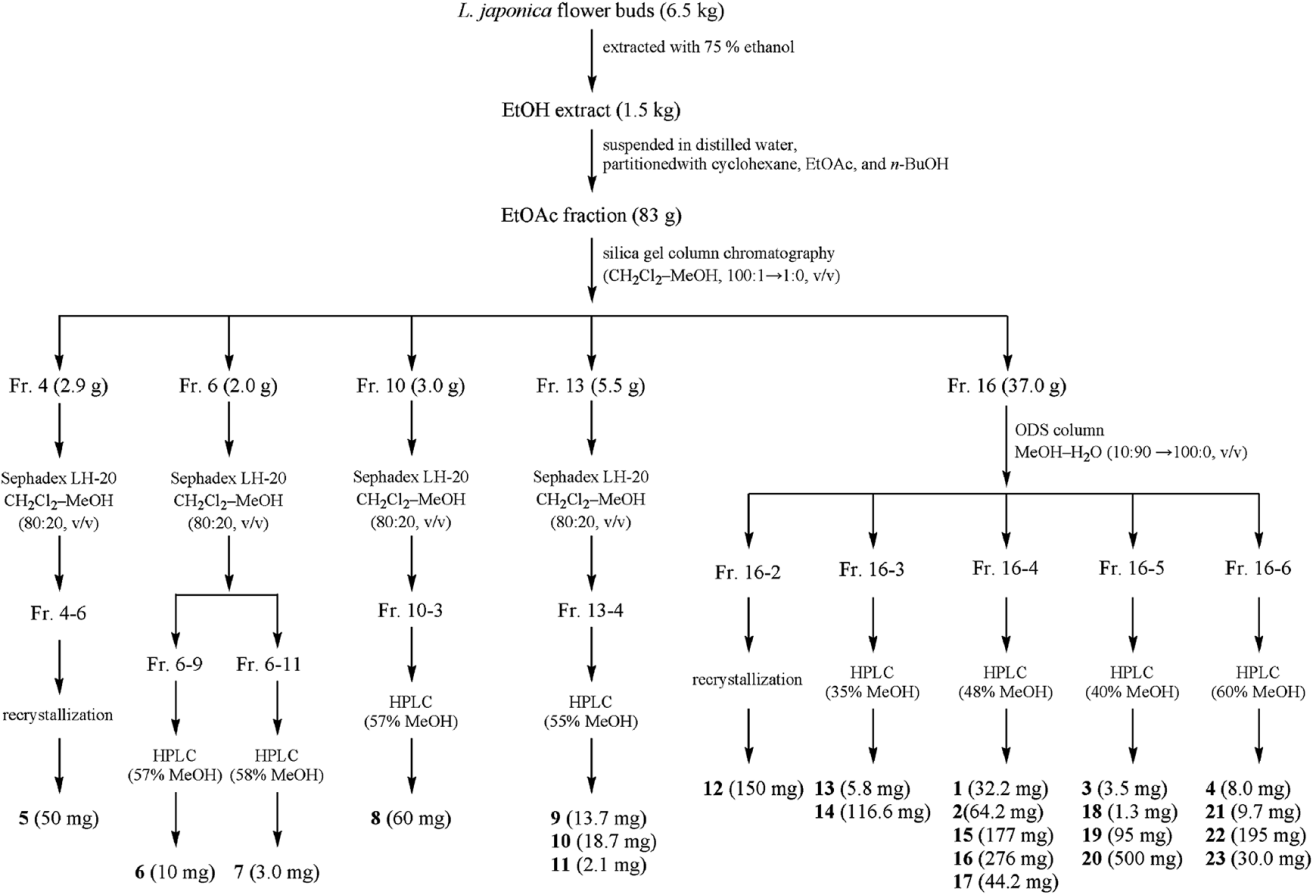
Extraction and isolation procedure of caffeoylquinic acid derivatives from *L. japonica* flower buds.

Fr. 16 (yellow colloidal, 37.0 g), was chromatographed on an ODS column (5.5 × 28 cm) gradually eluted with MeOH–H_2_O (10 : 90 → 100 : 0, v/v) to obtain 9 subfractions (Fr. 16–1 to Fr. 16–9). Compound 12 (150 mg) was obtained by recrystallizing in methanol from Fr. 16–2. Fr. 16–3 was subjected to preparative HPLC (Cosmosil 5C_18_-MS-II, 5 μm, 20 × 250 mm, flow rate: 8 ml min^−1^, wave length: 330 nm, MeOH : H_2_O, 35 : 65, v/v) yielding compound 13 (5.8 mg, *t*_R_: 28.2 min) and compound 14 (116.6 mg, *t*_R_: 36.6 min). Fr. 16–4 was purified by preparative HPLC (Cosmosil 5C_18_-MS-II, 5 μm, 20 × 250 mm, flow rate: 8 ml min^−1^, wave length: 330 nm) using MeOH–H_2_O (48 : 52, v/v) as the eluent to give compound 15 (177 mg, *t*_R_: 9.8 min), compound 16 (276 mg, *t*_R_: 10.8 min), compound 1 (32.2 mg, *t*_R_: 13.9 min), compound 2 (64.2 mg, *t*_R_: 16.6 min), and compound 17 (44.2 mg, *t*_R_: 21.9 min). Fr. 16–5 was separated by preparative HPLC (Cosmosil 5C_18_-MS-II, 5 μm, 20 × 250 mm, flow rate: 8 ml min^−1^, wave length: 330 nm, MeOH : H_2_O, 40 : 60, v/v) to afford compound 18 (1.3 mg, *t*_R_: 32.9 min), compound 19 (95 mg, *t*_R_: 39.3 min), compound 3 (3.5 mg, *t*_R_: 54.1 min) and compound 20 (500 mg, *t*_R_: 59.2 min). Next, Fr. 16–6 was subjected to preparative HPLC (Cosmosil 5C_18_-MS-II, 5 μm, 20 × 250 mm, flow rate: 8 ml min^−1^, MeOH : H_2_O, 60 : 40, v/v) yielding compound 21 (9.7 mg, *t*_R_: 7.6 min), compound 22 (195 mg, *t*_R_: 10.0 min), compound 4 (8.0 mg, *t*_R_: 13.1 min) and compound 23 (30.0 mg, *t*_R_: 14.5 min). Fig. 2 shows the extraction and isolation procedure of caffeoylquinic acid derivatives from the flower buds of *L. japonica*.

#### 4-*O-trans*-Caffeoyl-5-*O*-[(3*S*)-3-hydroxy-3-(3,4-dihydroxyphenyl)-propionyl] quinic acid methyl ester (1)

Pale yellow, amorphous powder; HR-ESI-MS *m*/*z* 547.1627 [M − H]^−^; ^1^H NMR (400 MHz, d_6_-DMSO) and ^13^C NMR (100 MHz, d_6_-DMSO) spectrum information, see Table 1.

**Table 1:** ^1^H NMR (400 MHz) and ^13^C NMR (100 MHz) data of Compounds 1–4 in *d*_6_-DMSO

Position	Compound 1		Compound 2		Compound 3		Compound 4	
	*δ*_H_	*δ*_C_	*δ*_H_	*δ*_C_	*δ*_H_	*δ*_C_	*δ*_H_	*δ*_C_
1		72.90		72.62		72.27		76.80
2	1.98 (m); 2.16 (d, 11.0)	35.46	1.97 (m); 2.16 (m)	34.97	1.98 (m); 2.20 (m)	34.51	2.01 (m); 2.21 (m)	37.58
3	3.76 (s)	67.47	3.80 (s)	66.86	3.86 (s)	66.12	5.26 (d, 3.9)	67.53
4	5.03 (m)	71.07	5.07 (m)	71.07	5.04 (d, 3.6),	70.90	4.93 (m)	73.25
5	5.14 (m)	70.33	5.07 (m)	70.29	5.12 (d, 10.0),	69.70	4.09 (d, 7.7)	65.46
6	1.98 (m)	34.82	1.97 (m); 2.16 (m)	34.47	1.98 (m); 2.20 (m)	34.24	1.88 (m); 2.21 (m)	36.08
7		174.10		173.88		173.59		173.23
7–OCH_3_	3.60 (s)	52.11	3.59 (d, 3.2)	52.09	3.59 (s)	51.80	3.60 (s)	51.91
1′		125.89		125.57		125.93		125.73
2′	7.05 (s)	115.30	7.06 (s)	113.97	7.05 (s)	114.92	7.04 (d, 2.0)	114.88
3′		145.80		145.18		145.35		144.61
4′		148.49		148.77		148.55		147.60
5′	6.77 (d, 6.8)	116.74	6.79 (d, 6.8)	115.60	6.78 (d, 8.0)	115.82	6.75 (d, 8.0)	115.79
6′	7.00 (d, 8.0)	121.39	7.00 (d, 8.0)	121.59	7.00 (dd, 2.0, 8.0)	121.33	6.98 (dd, 2.0, 8.0)	121.36
7′	7.49 (d, 15.9)	145.04	7.42 (d, 15.9)	145.61	7.43 (d, 15.9)	145.60	7.50 (d, 15.9)	145.58
8′	6.24 (d, 15.9)	114.95	6.13 (d, 15.9)	113.87	6.12 (d, 15.9)	113.51	6.27 (d, 15.9)	113.85
9′		166.21		165.47		165.13		165.88
1′′		135.83		131.65		125.21		125.44
2′′	6.75 (s)	113.51	6.72 (s)	114.90	7.45 (s)	117.96	7.42 (d, 2.0)	117.91
3′′		144.58		145.44		144.47		144.82
4′′		145.15		145.86		147.29		148.47
5′′	6.65 (d, 8.0)	116.06	6.71 (d, 8.0)	116.13	6.72 (d, 8.0)	114.55	6.72 (d, 8.0)	115.00
6′′	6.58 (m)	117.00	6.60 (d, 8.0)	118.05	7.12 (dd, 2.0, 8.0)	123.74	7.07 (dd, 2.0, 8.0)	123.88
7′′	4.76 (m)	69.35	4.42 (m)	79.24	6.78 (d, 12.8)	143.63	6.80 (d, 12.8)	145.48
8′′	2.47 (m, 5.0); 2.50 (m, 5.0)	44.84	2.57 (dd, 8.0, 5.0); 2.67 (dd, 8.0, 5.0)	43.20	5.76 (d, 12.8)	115.56	5.58 (d, 12.8)	114.12
9′′		169.87		169.93		165.13		164.61
7′′–OCH_3_			3.07 (s)	55.91				

#### 4-*O-trans*-Caffeoyl-5-*O*-[(3*S*)-3-methoxy-3-(3,4-dihydroxyphenyl)-propionyl] quinic acid methyl ester (2)

Pale yellow, amorphous powder; HR-ESI-MS *m*/*z* 561.1877 [M − H]^−^; ^1^H NMR (400 MHz, d_6_-DMSO) and ^13^C NMR (100 MHz, d_6_-DMSO) spectrum information, see Table 1.

#### 4-*O-trans*-Caffeoyl-5-*O-cis*-caffeoyl quinic acid methyl ester (3)

Pale yellow, amorphous powder; HR-ESI-MS *m*/*z* 529. 1506 [M − H]^−^; HR-ESI-MS^2^*m*/*z* 367.1129 [M–caffeoyl]^−^, 161.0289 [caffeoyl]^−^; ^1^H NMR (400 MHz, d_6_-DMSO) and ^13^C NMR (100 MHz, d_6_-DMSO) spectrum information, see Table 1.

#### 3-*O-cis*-Caffeoyl-4-*O-trans*-caffeoyl quinic acid methyl ester (4)

Pale yellow, amorphous powder; HR-ESI-MS *m*/*z* 529.1573 [M − H]^−^; HR-ESI-MS *m*/*z* 529. 1506 [M − H]^−^; HR-ESI-MS^2^*m*/*z* 367.1185 [M–caffeoyl]^−^, 161.0295 [caffeoyl]^−^; ^1^H NMR (400 MHz, d_6_-DMSO) and ^13^C NMR (100 MHz, d_6_-DMSO) spectrum information, see Table 1.

#### Isovanillic acid (5)

Pale yellow, amorphous powder; ^1^H NMR (400 MHz, d_6_-DMSO) *δ*_H_: 7.43 (2H, m, H-2, 6), 6.82 (1H, d, *J* = 8.6 Hz, H-5), 3.79 (3H, s, H–OCH_3_); ^13^C NMR (100 MHz, d_6_-DMSO) *δ*_C_: 121.68 (C-1), 115.06 (C-2), 151.13 (C-3), 147.25 (C-4), 112.82 (C-5), 123.49 (C-6), 167.22 (C-7), 55.60 (–OCH_3_).

#### Caffeic acid ethyl ester (6)

Pale yellow, amorphous powder; ^1^H NMR (400 MHz, d_6_-DMSO) *δ*_H_: 7.47 (1H, d, *J* = 15.9 Hz, H-7), 7.05 (1H, s, H-2), 7.00 (1H, d, *J* = 8.0, H-6), 6.76 (1H, d, *J* = 8.0 Hz, H-5), 6.25 (1H, d, *J* = 15.9 Hz, H-8), 4.15 (2H, q, *J* = 7.1 Hz, H-10), 1.24 (3H, t, *J* = 7.1 Hz, H-11); ^13^C NMR (100 MHz, d_6_-DMSO) *δ*_C_: 125.47 (C-1), 114.77 (C-2), 148.34 (C-3), 145.53 (C-4), 115.69 (C-5), 121.29 (C-6), 144.95 (C-7), 114.01 (C-8), 166.50 (C-9), 59.64 (C-10), 14.22 (C-11).

#### 3,4-Dihydroxybenzaldehyde (7)

Pale yellow, amorphous powder; ^1^H NMR (400 MHz, d_6_-DMSO) *δ*_H_: 9.70 (1H, s, H-7), 7.27 (1H, d, *J* = 2.0 Hz, H-6), 7.23 (1H, s, H-2), 6.90 (1H, d, *J* = 8.6 Hz, H-5); ^13^C NMR (100 MHz, d_6_-DMSO) *δ*_C_: 124.33 (C-1), 115.45 (C-2), 152.10 (C-3), 145.82 (C-4), 114.33 (C-5), 128.78 (C-6), 190.93 (C-7).

#### Caffeic acid (8)

Pale yellow, amorphous powder; ^1^H NMR (400 MHz, d_6_-DMSO) *δ*_H_: 7.54 (1H, d, *J* = 15.9 Hz, H-7), 7.15 (1H, s, H-2), 7.04 (1H, d, *J* = 8.0, H-6), 6.86 (1H, d, *J* = 8.0 Hz, H-5), 6.29 (1H, d, *J* = 15.9 Hz, H-8); ^13^C NMR (100 MHz, d_6_-DMSO) *δ*_C_: 127.12 (C-1), 117.13 (C-2), 149.38 (C-3), 146.83 (C-4), 115.99 (C-5), 122.55 (C-6), 145.98 (C-7), 116.51 (C-8), 169.34 (C-9).

#### 4-*O*-Caffeoylquinic acid ethyl ester (9)

Yellow, amorphous powder; ^1^H NMR (400 MHz, d_6_-DMSO) *δ*_H_: 7.39 (1H, d, *J* = 15.9 Hz, H-7′), 7.02 (1H, s, H-2′), 6.96 (1H, d, *J* = 8.0, H-6′), 6.77 (1H, d, *J* = 8.0 Hz, H-5′), 6.11 (1H, d, *J* = 15.9 Hz, H-8′), 5.02 (1H, d, *J* = 3.7 Hz, H-4), 4.00 (2H, m, H-8), 3.89 (1H, m, H-5), 3.58 (1H, m, H-3), 2.11 (2H, m, H-2a, 6a), 1.93 (1H, m, H-2b), 1.77 (2H, m, H-6b), 1.12 (3H, t, d, *J* = 7.1 Hz, H-9); ^13^C NMR (100 MHz, d_6_-DMSO) *δ*_C_: 73.08 (C-1), 35.16 (C-2), 67.00 (C-3), 71.04 (C-4), 69.50 (C-5), 37.16 (C-6), 173.06 (C-7), 60.27 (C-8), 13.79 (C-9), 125.37 (C-1′), 115.84 (C-2′), 148.47 (C-3′), 145.62 (C-4′), 114.55 (C-5′), 121.28 (C-6′), 145.04 (C-7′), 113.85 (C-8′), 165.40 (C-9′).

#### 4,5-Di-*O*-caffeoylquinic acid ethyl ester (10)

Yellow, amorphous powder; ^1^H NMR (400 MHz, d_6_-DMSO) *δ*_H_: 7.50 (1H, d, *J* = 15.9 Hz, H-7′), 7.43 (1H, d, *J* = 15.9 Hz, H-7′′), 7.04 (2H, s, H-2′,2′′), 6.99 (2H, t, *J* = 8.0, 8.0 Hz, H-6′,6′′), 6.78 (2H, d, *J* = 5.1 Hz, H-5′,5′′), 6.26 (1H, d, *J* = 15.9 Hz, H-8′), 6.13 (1H, d, *J* = 15.9 Hz, H-8′′), 5.19 (1H, d, *J* = 9.4 Hz, H-5), 5.09 (1H, s, H-4), 4.05 (2H, m, H-8), 3.85 (1H, s, H-3), 2.20 (2H, dd, *J* = 3.8, 13.1 Hz, H-2, 6), 2.01 (2H, d, *J* = 12.3 Hz, H-2, 6), 1.15 (3H, t, d, *J* = 7.1 Hz, H-9); ^13^C NMR (100 MHz, d_6_-DMSO) *δ*_C_: 72.43 (C-1), 34.40 (C-2), 66.70 (C-3), 70.96 (C-4), 69.95 (C-5), 33.25 (C-6), 174.30 (C-7), 60.41 (C-8), 13.79 (C-9), 125.61 (C-1′), 125.33 (C-1′′), 115.89 (C-2′), 115.81 (C-2′′), 148.56 (C-3′), 148.31 (C-3′′), 145.31 (C-4′), 144.93 (C-4′′), 113.65 (C-5′, 5′′), 121.32 (C-6′), 121.20 (C-6′′), 145.65 (C-7′), 145.58 (C-7′′), 114.77 (C-8′), 114.62 (C-8′′), 165.99 (C-9′), 165.29 (C-9′′).

#### 1,4-Di-*O*-caffeoylquinic acid (11)

Yellow, amorphous powder; ^1^H NMR (400 MHz, d_6_-DMSO) *δ*_H_: 7.62 (1H, d, *J* = 15.9 Hz, H-7′), 7.47 (1H, d, *J* = 15.9 Hz, H-7′′), 7.10 (1H, s, H-2′), 7.06 (1H, d, *J* = 8.0 Hz, H-6′), 7.02 (1H, s, H-2′′), 6.97 (1H, d, *J* = 8.0 Hz, H-6′′), 6.78 (2H, d, *J* = 8.0 Hz, H-5′,5′′), 6.32 (1H, d, *J* = 15.9 Hz, H-8′), 6.13 (1H, d, *J* = 15.9 Hz, H-8′′), 5.15 (1H, d, *J* = 4.8 Hz, H-4), 4.99 (1H, d, *J* = 3.5 Hz, H-3), 4.90 (1H, d, *J* = 4.7 Hz, H-5), 2.34 (1H, d, *J* = 11.8 Hz, H-2a), 2.01 (1H, d, *J* = 14.2 Hz, H-6a), 1.24 (1H, s, H-2b), 1.17 (1H, d, *J* = 6.7 Hz, H-6b); ^13^C NMR (100 MHz, d_6_-DMSO) *δ*_C_: 72.95 (C-1), 38.56 (C-2), 66.67 (C-3), 70.68 (C-4), 69.17 (C-5), 37.78 (C-6), 177.55 (C-7), 125.33 (C-1′), 125.22 (C-1′′), 115.88 (C-2′), 115.77 (C-2′′), 148.88 (C-3′), 148.77 (C-3′′), 146.82 (C-4′), 146.32 (C-4′′), 113.06 (C-5′), 112.67 (C-5′′), 121.80 (C-6′), 121.49 (C-6′′), 145.67 (C-7′), 145.64 (C-7′′), 115.05 (C-8′), 114.78 (C-8′′), 164.88 (C-9′), 164.68 (C-9′′).

#### 3-*O*-Caffeoylquinic acid (12)

Pale yellow, amorphous powder; ^1^H NMR (400 MHz, d_6_-DMSO) *δ*_H_: 7.45 (1H, d, *J* = 15.9 Hz, H-7′), 7.07 (1H, s, H-2′), 7.01 (1H, d, *J* = 8.0, H-6′), 6.80 (1H, d, *J* = 8.0 Hz, H-5′), 6.18 (1H, d, *J* = 15.9 Hz, H-8′), 5.10 (1H, m, H-3), 3.96 (1H, m, H-5), 3.60 (1H, dd, *J* = 6.9, 2.5 Hz, H-4), 2.02 (3H, m, H-2, 6a), 1.82 (1H, m, H-6b); ^13^C NMR (100 MHz, d_6_-DMSO) *δ*_C_: 74.85 (C-1), 37.63 (C-2), 71.75 (C-3), 72.20 (C-4), 69.47 (C-5), 38.52 (C-6), 176.35 (C-7), 126.92 (C-1′), 117.09 (C-2′), 149.66 (C-3′), 146.87 (C-4′), 116.08 (C-5′), 122.72 (C-6′), 146.29 (C-7′), 115.61 (C-8′), 167.09 (C-9′).

#### 1-*O*-Caffeoylquinic acid methyl ester (13)

Pale yellow, amorphous powder; ^1^H NMR (400 MHz, d_6_-DMSO) *δ*_H_: 7.49 (1H, d, *J* = 15.9 Hz, H-7′), 7.04 (1H, s, H-2′), 7.00 (1H, d, *J* = 8.0, H-6′), 6.77 (1H, d, *J* = 8.0 Hz, H-5′), 6.27 (1H, d, *J* = 15.9 Hz, H-8′), 5.01 (1H, s, H-4), 4.86 (1H, d, *J* = 5.0 Hz, H-5), 4.72 (1H, dd, *J* = 6.8, 3.0 Hz, H-3), 3.61 (3H, s, H–OCH_3_), 2.09 (1H, dd, *J* = 13.0, 3.0 Hz, H-2a), 1.98 (1H, dd, *J* = 13.0, 7.0 Hz, H-6a), 1.83 (2H, m, H-2b, 6b); ^13^C NMR (100 MHz, d_6_-DMSO) *δ*_C_: 75.98 (C-1), 38.20 (C-2), 64.58 (C-3), 73.82 (C-4), 65.74 (C-5), 38.20 (C-6), 174.01 (C-7), 51.87 (–OCH_3_), 125.83 (C-1′), 116.04 (C-2′), 148.54 (C-3′), 145.82 (C-4′), 114.97 (C-5′), 121.42 (C-6′), 145.05 (C-7′), 114.81 (C-8′), 166.43 (C-9′).

#### 4-*O*-Caffeoylquinic acid methyl ester (14)

Pale yellow, amorphous powder; ^1^H NMR (400 MHz, d_6_-DMSO) *δ*_H_: 7.40 (1H, d, *J* = 15.9 Hz, H-7′), 7.04 (1H, s, H-2′), 6.96 (1H, d, *J* = 8.0, H-6′), 6.78 (1H, d, *J* = 8.0 Hz, H-5′), 6.13 (1H, d, *J* = 15.9 Hz, H-8′), 5.05 (1H, d, *J* = 3.2 Hz, H-4), 3.92 (1H, s, H-5), 3.61 (1H, s, H-3), 3.56 (3H, s, –OCH_3_), 2.10 (2H, m, H-2), 1.97 (1H, m, H-6a), 1.81 (2H, m, H-6b); ^13^C NMR (100 MHz, d_6_-DMSO) *δ*_C_: 73.47 (C-1), 35.61 (C-2), 67.44 (C-3), 71.14 (C-4), 69.84 (C-5), 37.38 (C-6), 173.82 (C-7), 52.00 (C–OCH_3_), 125.66 (C-1′), 116.08 (C-2′), 148.63 (C-3′), 145.79 (C-4′), 114.76 (C-5′), 121.58 (C-6′), 145.32 (C-7′), 114.17 (C-8′), 165.68 (C-9′).

#### 3-*O*-Caffeoylquinic acid methyl ester (15)

Pale yellow, amorphous powder; ^1^H NMR (400 MHz, d_6_-DMSO) *δ*_H_: 7.42 (1H, d, *J* = 15.9 Hz, H-7′), 7.06 (1H, s, H-2′), 6.95 (1H, d, *J* = 8.0, H-6′), 6.79 (1H, d, *J* = 8.0 Hz, H-5′), 6.16 (1H, d, *J* = 15.9 Hz, H-8′), 5.11 (1H, d, *J* = 3.6 Hz, H-3), 3.98 (1H, s, H-5), 3.64 (1H, s, H-4), 3.57 (3H, s, –OCH_3_), 2.06 (3H, m, H-2, 6a), 1.86 (1H, m, H-6b); ^13^C NMR (100 MHz, d_6_-DMSO) *δ*_C_: 74.14 (C-1), 36.34 (C-2), 70.62 (C-3), 71.34 (C-4), 68.31 (C-5), 37.60 (C-6), 174.17 (C-7), 52.37 (–OCH_3_), 126.15 (C-1′), 116.42 (C-2′), 148.86 (C-3′), 146.05 (C-4′), 115.01 (C-5′), 122.00 (C-6′), 145.66 (C-7′), 114.61 (C-8′), 166.24 (C-9′).

#### 4,5-Di-*O*-caffeoylquinic acid (16)

Pale yellow, amorphous powder; ^1^H NMR (400 MHz, d_6_-DMSO) *δ*_H_: 7.56 (1H, d, *J* = 15.9 Hz, H-7′), 7.52 (1H, d, *J* = 15.9 Hz, H-7′′),7.12 (2H, s, H-2′, 2′′), 6.97 (2H, s, H-6′, 6′′), 6.82 (2H, s, H-5′, 5′′), 6.32 (1H, d, *J* = 15.9 Hz, H-8′), 6.24 (1H, d, *J* = 15.9 Hz, H-8′′), 5.34 (1H, s, H-5), 5.29 (1H, s, H-4), 3.96 (1H, s, H-3), 2.25 (2H, s, H-2, 6), 2.11 (2H, s, H-2, 6); ^13^C NMR (100 MHz, d_6_-DMSO) *δ*_C_: 73.63 (C-1), 36.66 (C-2), 68.95 (C-3), 71.57 (C-4), 71.19 (C-5), 35.40 (C-6), 176.25 (C-7), 126.74 (C-1′), 126.67 (C-1′′), 116.65 (C-2′, 2′′), 148.95 (C-3′), 148.84 (C-3′′), 146.18 (C-4′, 4′′), 115.61 (C-5′), 115.43 (C-5′′), 122.49 (C-6), 122.32 (C-6′′), 146.07 (C-7′), 145.76 (C-7′′), 115.27 (C-8′), 115.07 (C-8′′), 167.23 (C-9′), 166.75 (C-9′′).

#### Caffeic acid methyl ester (17)

Pale yellow, amorphous powder; HR-ESI-MS *m*/*z* 193.0540 [M − H]^−^; ^1^H NMR (400 MHz, d_6_-DMSO) *δ*_H_: 7.49 (1H, d, *J* = 15.9 Hz, H-7), 7.07 (1H, s, H-2), 7.00 (1H, d, *J* = 8.0 Hz, H-6), 6.78 (1H, d, *J* = 8.0 Hz, H-5), 6.27 (1H, d, *J* = 15.9 Hz, H-8), 3.68 (3H, s, H-10).

#### 5-*O*-Caffeoylquinic acid methyl ester (18)

Pale yellow, amorphous powder; ^1^H NMR (400 MHz, d_6_-DMSO) *δ*_H_: 7.48 (1H, d, *J* = 15.9 Hz, H-7′), 7.03 (1H, s, H-2′), 6.99 (1H, d, *J* = 8.0, H-6′), 6.76 (1H, d, *J* = 8.0 Hz, H-5′), 6.23 (1H, d, *J* = 15.9 Hz, H-8′), 5.13 (1H, m, H-5), 4.33 (1H, s, H-3), 3.76 (1H, s, H-4), 3.67 (3H, s, –OCH_3_), 2.18 (1H, m, H-6a), 2.09 (1H, m, H-6b), 1.97 (2H, m, H-2).

#### 3,4-Di-*O*-caffeoylquinic acid (19)

Pale yellow, amorphous powder; HR-ESI-MS *m*/*z* 515.1276 [M − H]^−^; ^1^H NMR (400 MHz, d_6_-DMSO) *δ*_H_: 7.52 (1H, d, *J* = 15.9 Hz, H-7′), 7.46 (1H, d, *J* = 15.9 Hz, H-7′′), 7.05 (2H, s, H-2′, 2′′), 6.98 (2H, s, H-6′, 6′′), 6.77 (2H, d, *J* = 8.0 Hz, H-5′, 5′′), 6.27 (1H, d, *J* = 15.9 Hz, H-8′), 6.18 (1H, d, *J* = 15.9 Hz, H-8′′), 5.42 (1H, s, H-3), 5.02 (1H, d, *J* = 6.7 Hz, H-4), 4.23 (1H, s, H-5), 2.20 (2H, d, *J* = 11.9 Hz, H-2), 2.09 (2H, d, *J* = 10.4 Hz, H-6a), 1.96 (1H, d, *J* = 6.1 Hz, H-6b); ^13^C NMR (100 MHz, d_6_-DMSO) *δ*_C_: 74.03 (C-1), 37.82 (C-2), 68.00 (C-3), 73.77 (C-4), 66.83 (C-5), 37.58 (C-6), 175.09 (C-7), 125.87 (C-1′, 1′′), 116.22 (C-2′), 116.16 (C-2′′), 148.80 (C-3′, 3′′), 145.92 (C-4′, 4′′), 115.18 (C-5′), 115.15 (C-5′′), 121.90 (C-6′), 121.82 (C-6′′), 145.89 (C-7′, 7′′), 114.28 (C-8′), 114.05 (C-8′′), 166.38 (C-9′), 165.98 (C-9′′).

#### 4,5-Di-*O*-caffeoylquinic acid methyl ester (20)

Pale yellow, amorphous powder; ^1^H NMR (400 MHz, d_6_-DMSO) *δ*_H_: 7.56 (1H, d, *J* = 15.9 Hz, H-7′), 7.49 (1H, d, *J* = 15.9 Hz, H-7′′), 7.10 (2H, s, H-2′, 2′′), 6.98 (2H, s, H-6′, 6′′), 6.81 (2H, s, H-5′, 5′′), 6.31 (1H, d, *J* = 15.9 Hz, H-8′), 6.20 (1H, d, *J* = 15.9 Hz, H-8′′), 5.28 (1H, s, H-5), 5.21 (1H, s, H-4), 3.94 (1H, s, H-3), 3.61 (3H, s, –OCH_3_), 2.25 (2H, s, H-2, 6), 2.10 (2H, s, H-2, 6); ^13^C NMR (100 MHz, d_6_-DMSO) *δ*_C_: 73.27 (C-1), 35.77 (C-2), 67.91 (C-3), 71.33 (C-4), 70.74 (C-5), 35.03 (C-6), 174.30 (C-7), 52.38 (C–OCH_3_), 126.34 (C-1′), 126.08 (C-1′′), 116.45 (C-2′), 116.38 (C-2′′), 148.89 (C-3′), 148.67 (C-3′′), 146.04 (C-4′), 145.98 (C-4′′), 115.09 (C-5′, 5′′), 121.98 (C-6′), 121.89 (C-6′′), 145.85 (C-7′), 145.46 (C-7′′), 115.21 (C-8′), 114.41 (C-8′′), 166.69 (C-9′), 166.01 (C-9′′).

#### 3,5-Di-*O*-caffeoylquinic acid (21)

Pale yellow, amorphous powder; ^1^H NMR (400 MHz, d_6_-DMSO) *δ*_H_: 7.50 (1H, d, *J* = 15.9 Hz, H-7′), 7.43 (1H, d, *J* = 15.9 Hz, H-7′′),7.05 (2H, s, H-2′, 2′′), 7.10 (2H, d, *J* = 8.0 Hz, H-6′, 6′′), 6.77 (2H, m, H-5′, 5′′), 6.26 (1H, d, *J* = 15.9 Hz, H-8′), 6.14 (1H, d, *J* = 15.9 Hz, H-8′′), 5.80 (1H, s, H-3), 5.41 (1H, s, H-5), 3.86 (1H, s, H-4), 3.59 (3H, s, H–OCH_3_), 2.21 (2H, m, H-2, 6), 2.01 (2H, m, H-2, 6).

#### 3,4-Di-*O*-caffeoylquinic acid methyl ester (22)

Pale yellow, amorphous powder; ^1^H NMR (400 MHz, d_6_-DMSO) *δ*_H_: 7.56 (1H, d, *J* = 15.9 Hz, H-7′), 7.47 (1H, d, *J* = 15.9 Hz, H-7′′),7.09 (2H, s, H-2′,2′′), 6.97 (2H, s, H-6′,6′′), 6.80 (2H, d, *J* = 8.0 Hz, H-5′,5′′), 6.31 (1H, d, *J* = 15.9 Hz, H-8′), 6.19 (1H, d, *J* = 15.9 Hz, H-8′′), 5.42 (1H, s, H-3), 5.06 (1H, d, *J* = 6.7 Hz, H-4), 4.25 (1H, s, H-5), 3.61 (3H, s, –OCH_3_), 2.20 (2H, s, H-2), 2.14 (1H, s, H-6a), 2.00 (1H, s, H-6b); ^13^C NMR (100 MHz, d_6_-DMSO) *δ*_C_: 74.16 (C-1), 37.93 (C-2), 68.10 (C-3), 73.32 (C-4), 66.60 (C-5), 37.23 (C-6), 173.86 (C-7), 52.50 (–OCH_3_), 126.09 (C-1′), 125.95 (C-1′′), 116.35 (C-2′,2′′), 148.94 (C-3′), 148.84 (C-3′′), 146.15 (C-4′), 146.07 (C-4′′), 115.11 (C-5′, 5′′), 122.06 (C-6′, 6′′), 145.98 (C-7′, 7′′), 114.39 (C-8′), 113.99 (C-8′′), 166.55 (C-9′), 165.99 (C-9′′).

#### 3,4,5-Tri-*O*-caffeoylquinic acid methyl ester (23)

Yellow, amorphous powder; ^1^H NMR (400 MHz, d_6_-DMSO) *δ*_H_: 7.48 (1H, d, *J* = 15.9 Hz, H-7′, 7′′), 7.45 (1H, d, *J* = 15.9 Hz, H-7′′′),7.04 (3H, s, H-2′, 2′′, 2′′′), 6.97 (3H, m, H-6′, 6′′, 6′′′), 6.75 (3H, m, H-5′, 5′′, 5′′′), 6.25 (1H, d, *J* = 15.9 Hz, H-8′), 6.23 (1H, d, *J* = 15.9 Hz, H-8′′), 6.16 (1H, d, *J* = 15.9 Hz, H-8′′′), 5.44 (1H, m, H-3), 5.42 (1H, s, H-5), 5.25 (1H, m, H-4), 3.64 (3H, s, –OCH_3_), 2.35 (2H, m, H-2, 6), 2.06 (2H, m, H-2, 6); ^13^C NMR (100 MHz, d_6_-DMSO) *δ*_C_: 72.52 (C-1), 38.89 (C-2), 67.68 (C-3), 69.45 (C-4), 67.75 (C-5), 39.10 (C-6), 173.61 (C-7), 52.04 (C–OCH_3_), 125.43 (C-1′), 125.29 (C-1′′), 125.22 (C-1′′′), 115.82 (C-2′), 115.77 (C-2′′), 115.71 (C-2′′′), 148.64 (C-3′), 148.58 (C-3′′), 148.43 (C-3′′′), 145.98 (C-4′), 145.87 (C-4′′), 145.48 (C-4′′′), 114.91 (C-5′), 114.83 (C-5′′, 5′′′), 121.56 (C-6), 121.44 (C-6′′), 121.36 (C-6′′′), 145.61 (C-7′), 145.56 (C-7′′, 7′′′), 113.86 (C-8′), 113.20 (C-8′′), 113.14 (C-8′′′), 165.74 (C-9′), 165.51 (C-9′′), 165.16 (C-9′′′).

### Ester hydrolysates and HPLC analysis

Ester hydrolysates of compounds 1–4 were carried out according to the following method. In brief, compounds 1–4 (each 2.0 mg) were hydrolysed by reaction in 1 N NaOH (4 ml) and tetrahydrofuran (4 ml) at 0 °C for 4 h. The reaction mixture was extracted by adjusting pH to 4.5. The extraction product was directly analysed by reversed-phase HPLC using a Diamonsil C_18_ analytical column (5 μm, 4.6 × 250 mm), which was eluted isocratically with 25% acetonitrile in water (containing 0.1% formic acid) at a flow rate of 1 ml min^−1^. The temperature of the column oven was 25 °C, and 20 μl was injected into the system each time. The UV spectra were collected at 327 nm. Peaks of the ester hydrolysate of compounds 1–4 were identified by comparing the retention times of authentic samples of 4-*O*-caffeoylquinic acid methyl ester (compound 14, *t*_R_ = 10.411 min) after simultaneous treatment under the same conditions.

### Cytotoxic activity assay

The HepG 2 cells and HepG 2.2.15 cells were from the American Type Culture Collection (ATCC, Manassas, USA). All the cells were maintained in DMEM containing 10% FBS (foetal bovine serum, HyClone, Logan, UT) and cultured at 37 °C (5% CO_2_, 95% relative humidity). The cytotoxicity assay was performed, according to the MTT method in 96-well microplates. Briefly, 200 μl of adherent cells was seeded into each well of the 96-well cell culture plates and allowed to adhere for 24 hours before drug addiction with an initial density of 1.0 × 10^5^ cells per ml. Each tumour cell line was exposed to the test compounds at concentrations of 100, 50, and 20 μg ml^−1^ (DMEM with 0.1% DMSO) for three times within 48 hours. The control group was treated in the same solvent. After 48 hours, the media and test samples were replaced with MTT (5 mg ml^−1^) and incubated in the dark for 4 h. The formazan crystals formed were dissolved in DMSO. Absorbance was measured using a microplate reader at 520 nm. Cell viability (%) was calculated as *A*_sample_/*A*_control_ × 100%.

### Anti-HBV activity assay

HepG 2.2.15 cells were maintained in DMEM containing 10% FBS (foetal bovine serum, HyClone, Logan, UT) and cultured at 37 °C (5% CO_2_, 95% relative humidity). The anti-HBV assay was performed according to previous research.^15^ Briefly, 500 μl of adherent cells was seeded into each well of the 24-well cell culture plates and allowed to adhere for 24 hours before drug addiction with an initial density of 3 × 10^5^ cells per mL. The cell supernatants were collected every three days and fresh cell culture medium contain corresponding test compounds were added. The experiment lasted nine days. The collected cell supernatants were used to analyse the levels of HBsAg, HBeAg and HBV DNA with their corresponding reagent kit. The levels of HBsAg and HBeAg in the supernatants were measured with an ELISA method. Real-time PCR assay was used to detect the HBV DNA. The relative level was calculated using the equation: relative level (%) = [(*A*_test_ − *A*_control_)/*A*_control_] × 100 (where *A* is the levels of secreted HBsAg or HBeAg or HBV DNA), and the subscript words ‘test’ and ‘control’ represent that the test sample and control group, respectively.

### Statistical analysis

All data is expressed as mean ± SD. At least three independent experiments were performed, each in quintuplicate. The data is analysed using a one-way ANOVA. Statistically significant effects were analysed, and the means are also compared using least-significant difference (LSD) test. Statistical significance is determined at *p* < 0.05.

## Results and discussion

### Structural identification of new caffeoylquinic acids

**Fig. 3:**
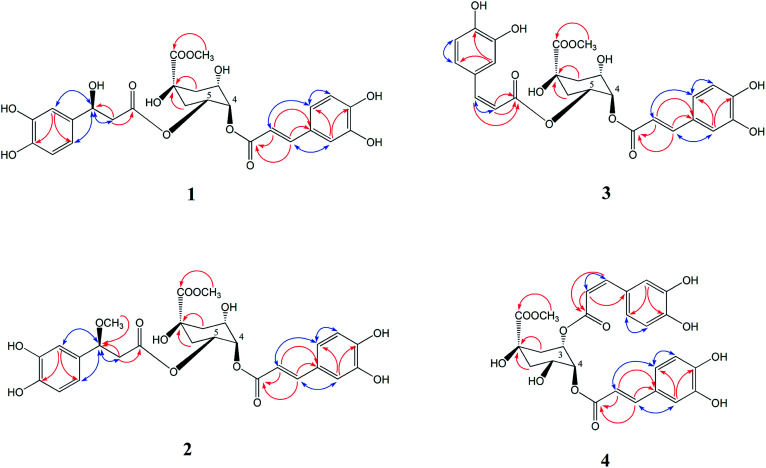
Key HMBC (H → C, red) and NOESY (H ↔ H, blue) correlations of novel compounds 1–4.

Compound 1 was isolated as a yellow amorphous powder, with the molecular formula of C_26_H_28_O_13_ as deduced from the [M − H]^−^ peak at *m*/*z* 547.1627 (calculated for C_26_H_27_O_13_, 547.1622) *via* HR-ESI-MS and supported by the ^13^C NMR spectral data. The ^1^H NMR and ^13^C NMR spectrum (Table 1) showed a 4,5-disubstituted quinic acid methyl ester derivative of compound 1, which was confirmed according to a methoxyl group signal at *δ*_H_ 3.60 (3H, m), three oxygen proton signals at *δ*_H_ 3.76 (1H, s), 5.03 (1H, m), 5.14 (1H, m), four proton signals at *δ*_H_ 1.98 (3H, m) and 2.16 (1H, d, *J* = 11.0 Hz) and eight carbon resonances at *δ*_C_ 72.90 (C-1), 35.46 (C-2), 67.47 (C-3), 71.07 (C-4), 70.33 (C-5), 34.82 (C-6), 174.10 (C-7), 52.11 (7–OCH_3_) by HSQC correlations analysis. An aromatic ABX system [*δ*_H_ 7.05 (1H, s), 7.00 (1H, d, *J* = 8.0 Hz), 6.77 (1H, d, *J* = 6.8 Hz)], together with a pair of coupled doublets [*δ*_H_ 7.49 (1H, d, *J* = 15.9 Hz) and 6.24 (1H, d, *J* = 15.9 Hz)], reveal the presence of a *trans*-caffeoyl moiety. Furthermore, another aromatic ABX system [*δ*_H_ 6.75 (1H, s), 6.65 (1H, d, *J* = 8.0 Hz), 6.58 (1H, m)] and three proton signals at *δ*_H_ 4.76 (1H, m), 2.50 (1H, t, *J* = 5.0 Hz), 2.47 (1H, t, *J* = 5.0 Hz) indicated the presence of a 3-hydroxy-3-(3,4-dihydroxyphenyl)-propionyl moiety, which was confirmed according to the observed NOESY correlations of *δ*_H_ 4.76 (H-7′′) with H-2′′ (*δ*_H_ 6.75), H-6′′ (*δ*_H_ 6.58) and H-8′′ (*δ*_H_ 2.47, 2.50), and HMBC correlations of *δ*_H_ 2.47, 2.50 (H-8′′) to *δ*_C_ 69.35 (C-7′′), 169.87 (C-9′′) in Fig. 3. Ester hydrolysates of compound 1 produced the corresponding 4-*O-trans*-caffeoylquinic acid methyl ester (14), which was identified by HPLC analysis. Therefore, we concluded that the *trans*-caffeoyl moiety was attached to C-4 of quinic acid methyl ester. Moreover, the other part of the ester hydrolysates 3-hydroxy-3-(3,4-dihydroxyphenyl)-propionic acid was measured to have an optical rotation value of [*α*]^22^_D_ = −4.6°, which was nearly equal to [*α*]^22^_D_ = −4.5° of β-d-glucopyranoside, 1′′-*O*-(7*S*)-7-(3-methoxyl-4-hydroxyphenyl)-7-methoxyethyl-3′′-α-l-rhamnopyranosyl-4′′-[(8*E*)-7-(3-methoxyl-4-hydroxyphenyl)-8-propenoate].^16^ Therefore, the C-7′′ configuration of compound 1 was identified as 7′′*S*. Consequently, compound 1 was determined as 4-*O-trans*-caffeoyl-5-*O*-[3*S*-hydroxy-3-(3,4-dihydroxyphenyl)-propionyl] quinic acid methyl ester.

Compound 2 was isolated as a yellow amorphous powder, and possessed the molecular formula of C_27_H_30_O_13_, based on HR-ESIMS at *m*/*z* 561.1877 (calculated for C_27_H_29_O_13_, 561.1873). The ^1^H and ^13^C NMR spectroscopic features (Table 1) were consistent with that of 1 but with a difference of one more methoxyl group at *δ*_H_ 3.07 (3H, s). The HMBC correlation from *δ*_H_ 3.07 to C-7′′ (*δ*_C_ 79.24) indicated that the extra methoxyl group was attached to C-7′′ of the propionyl moiety (Fig. 3). The 10 ppm down-field shift of C-7′′ in the ^13^C NMR spectrum further indicated the attachment of the extra methoxyl group to C-7′′. In a similar manner, the optical rotation of the ester hydrolysates of compound 2 was measured with value [*α*]^22^_D_ = −4.7°, which was nearly equal to [*α*]^22^_D_ = −4.6° of compound 1. Therefore, the C-7′′ configuration of compound 2 was also identified as 7′′S. Based on the above spectral evidences, the structure of compound 2 was elucidated as 4-*O-trans*-caffeoyl-5-*O*-[3*S*-methoxy-3-(3,4-dihydroxyphenyl)-propionyl] quinic acid methyl ester.

Compounds 3 and 4 were isolated as yellow amorphous powder. The molecular formula of compounds 3 and 4 were determined to be C_26_H_26_O_12_ by a negative HR-ESIMS molecule at *m*/*z* 529.1506 [M − H]^−^ and 529.1573 [M − H]^−^ (calculated for C_26_H_25_O_12_ 529.1531), respectively. The NMR data of compound 3 (Table 1) was similar to that of 4,5-di-*O*-caffeoylquinic acid methyl ester (20), except that the second pair of coupled doublet [*δ*_H_ 6.78 (1H, d, *J* = 12.8 Hz, H-7′′), 5.76 (1H, d, *J* = 12.8 Hz, H-8′′)] was different. This suggested compound 3 was also a 4,5-disubstituted quinic acid methyl ester but contains a *trans*-caffeoyl group and a *cis*-caffeoyl group. At the same time, a comparison of the ^1^H NMR and ^13^C NMR spectra of compound 4 (Table 1) with those of 3,4-di-*O*-caffeoylquinic acid methyl ester (22) proved that compound 4 was a 3,4-disubstituted quinic acid methyl ester contains a *trans*-caffeoyl group [*δ*_H_ 7.50 (1H, d, *J* = 15.9 Hz, H-7′), 7.04 (1H, d, *J* = 2.0 Hz, H-2′), 6.98 (1H, dd, *J* = 2.0, 8.0 Hz, H-6′), 6.75 (1H, d, *J* = 8.0 Hz, H-5′), 6.27 (1H, d, *J* = 15.9 Hz, H-8′)] and a *cis*-caffeoyl group [*δ*_H_ 7.42 (1H, d, *J* = 2.0 Hz, H-2′′), 7.07 (1H, dd, *J* = 2.0, 8.0 Hz, H-6′′), 6.72 (1H, d, *J* = 8.0 Hz, H-5′′), 6.80 (1H, d, *J* = 12.8 Hz, H-7′′), 5.58 (1H, d, *J* = 12.8 Hz, H-8′′)]. Ester hydrolysates of compounds 3 and 4 were all 4-*O-trans*-caffeoylquinic acid methyl ester (14), which were identified by HPLC analysis. The above results confirmed that the *cis*-caffeoyl group was linked to C-5 of compound 3 and C-3 of compound 4, respectively. The key HMBC and NOESY (H ↔ H, blue) correlations of compounds 3 and 4 were shown in Fig. 3. Therefore, the structures of compounds 3 and 4 were identified as 4-*O-trans*-caffeoyl-5-*O-cis*-caffeoyl quinic acid methyl ester and 3-*O-cis*-caffeoyl-4-*O-trans*-caffeoyl quinic acid methyl ester, respectively.

The other compounds were identified as isovanillic acid (5),^17^ caffeic acid ethyl ester (6),^18^ 3,4-dihydroxybenzaldehyde (7),^19^ caffeic acid (8),^20^ 4-*O*-caffeoylquinic acid ethyl ester (9),^21^ 4,5-di-*O*-caffeoylquinic acid ethyl ester (10),^22^ 1,4-di-*O*-caffeoylquinic acid (11),^23^ 3-*O*-caffeoylquinic acid (12),^20^ 1-*O*-caffeoylquinic acid methyl ester (13),^24^ 4-*O*-caffeoylquinic acid methyl ester (14),^25^ 3-*O*-caffeoylquinic acid methyl ester (15),^25^ 4,5-di-*O*-caffeoylquinic acid (16),^24,26^ caffeic acid methyl ester (17),^25^ 5-*O*-caffeoylquinic acid methyl ester (18) (Lu, Zheng, Wang, Ye, & Zhao, 2009),^24^ 3,4-di-*O*-caffeoylquinic acid (19),^24,26^ 4,5-di-*O*-caffeoylquinic acid methyl ester (20),^27^ 3,5-di-*O*-caffeoylquinic acid (21),^28^ 3,4-di-*O*-caffeoylquinic acid methyl ester (22),^29^ 3,4,5-tri-*O*-caffeoylquinic acid methyl ester (23).^30^

### Cytotoxicity

**Fig. 4:**
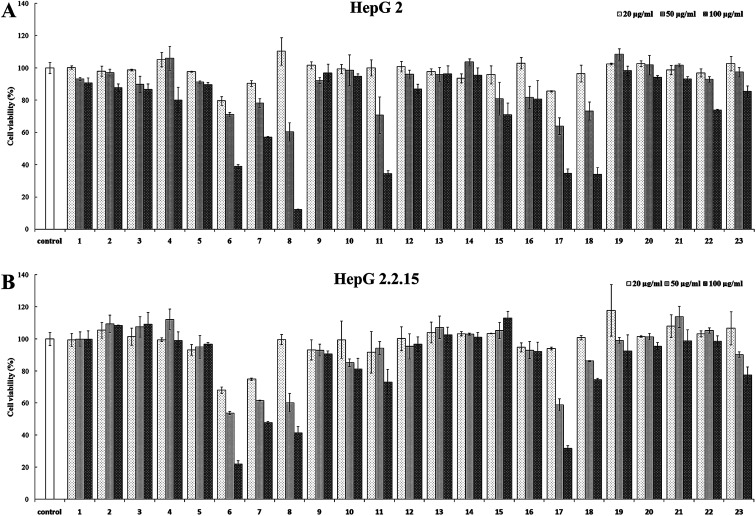
Cytotoxicity of compounds 1–23. (A) HepG 2 cell; (B) HepG 2.2.15 cell. Results are expressed as the mean ± SD (*n* = 3).

All the compounds (1–23) isolated were tested for their potential cytotoxicity against HepG 2 and HepG 2.2.15 cells. The purpose of this design was to determine their appropriate drug concentration in anti-HBV assay to eliminate the cytotoxicity interference. As shown in Fig. 4A and B, it was found that the cytotoxic results against HepG 2 were consistent with the experimental results of cytotoxicity against HepG 2.2.15 cells. This suggests that the effect of these caffeoylquinic acids on the growth of both two cell lines is consistent, although HepG 2.2.15 cells contain a complete HBV genome. The cytotoxicity assay results showed that, most compounds (1–5, 9–10, 12–16, 19–22) at a concentration range of 20–100 μg ml^−1^ showed no significant cytotoxicity against HepG 2 and HepG 2.2.15 cells. Therefore, the above compounds were selected 100 μg ml^−1^ for test drug concentration in the subsequent anti-HBV assay. In addition, compounds 11, 18 and 23 exhibit weak cytotoxicity against HepG 2 and HepG 2.2.15 cells at concentration of 100 μg ml^−1^ and selected 50 μg ml^−1^ for the next anti-HBV activities. At the same time, compounds 6–8 and 17 were selected 20 μg ml^−1^ due to their significantly cytotoxicity against HepG 2 and HepG 2.2.15 cells at 50 μg ml^−1^ concentration.

### Anti-HBV activities and their structure–activity relationship

**Fig. 5:**
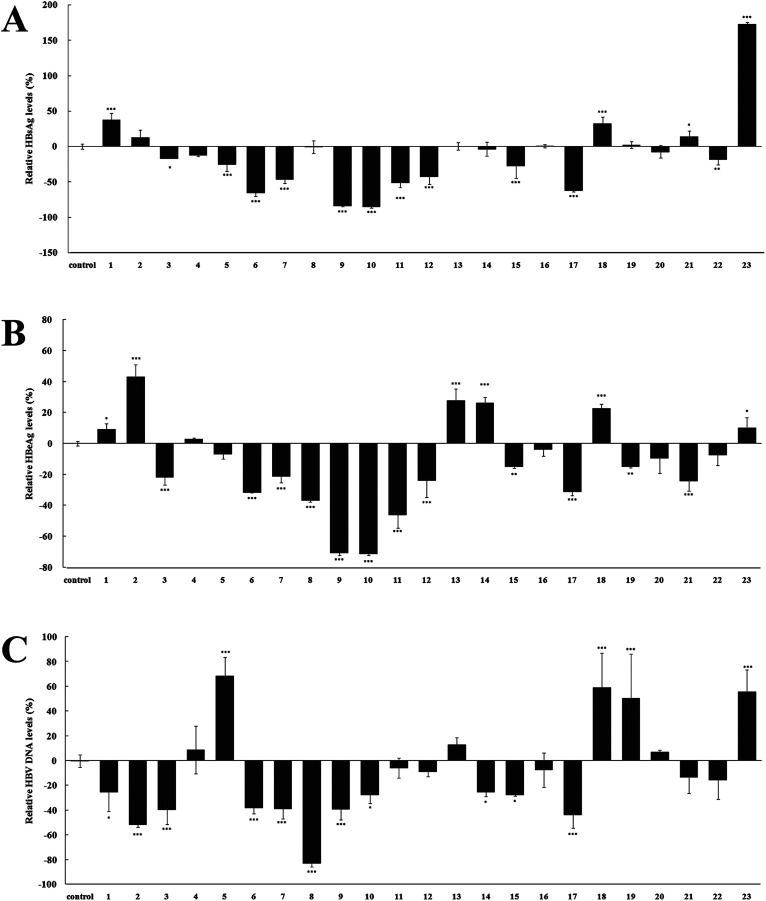
Anti-HBV activities of compounds 1–23. (A) Relative HBsAg levels; (B) relative HBeAg levels; (C) relative HBV DNA levels. The figure shows the results of the experiment on the sixth day. Compounds 6–8 and 17 were tested for 20 μg ml^−1^, compounds 11, 18 and 23 were tested for 50 μg ml^−1^, and the other compounds (1–5, 9–10, 12–16, 19–22) were tested for 100 μg ml^−1^. Results are expressed as the mean ± SD (*n* = 3). *p < 0.05 compared with the control group, **p < 0.01 compared with the control group, ***p < 0.001 compared with the control group.

To evaluate their anti-HBV activities, namely the inhibiting the secretion of Hepatitis B surface antigen (HBsAg) and Hepatitis B e antigen (HBeAg), as well as HBV DNA replication, compounds 1–23 were assayed on HepG 2.2.15 cell line stably transfected with the HBV genome *in vitro*, as reported previously.^15^ The experiment results are summarized in Fig. 5, and most caffeoylquinic acid derivatives showed excellent anti-HBV activities on HepG 2.2.15 cell. HBsAg is the outer protein of HBV, which is a sign of HBV infection. Its level is related to the numbers of HBV.^31,32^ As shown in Fig. 5A, compounds 5–7, 9–12, 15, 17 showed significant activities in inhibiting the secretion of HBsAg and their relative HBsAg levels were −25.53 ± 9.75, −65.67 ± 4.66, −46.27 ± 5.92, −83.82 ± 1.00, −84.97 ± 2.00, −50.87 ± 7.22, −42.55 ± 11.06, −27.66 ± 17.58, −61.94 ± 2.24%, respectively (*p* < 0.001). HBeAg is a major structural protein in the core of HBV. Its level indicates the different stages of the HBV infection, which is related to the replication rate of HBV.^31,33^ The HBeAg results of Fig. 5B showed that the replication rate of HBV was significantly inhibited by compounds 3, 6–12, 17, 21, especially compounds 9–11, and their relative HBeAg levels as low as −70.76 ± 1.46, −71.20 ± 1.22, −46.01 ± 8.51%, respectively (*p* < 0.001). While HBV DNA reflects the extents of HBV replication (Chen, *et al.*, 2006). As seen in Fig. 5C, most compounds exhibited remarkable decrease in the relative HBV DNA levels (*p* < 0.05). Compound 8 showed the most significant inhibitory activity and its relative HBV DNA level was decreased by 83.11 ± 2.75%. However, several compounds (4, 11, 12, 16, 20–22) showed no significantly inhibitory activity and some compounds (5, 18, 19, 23) showed strangely promoting activities. Unfortunately, we found that compound 23 promoted HBsAg and HBeAg secretion, and HBV DNA replication by 172.39 ± 2.59, 9.92 ± 6.91, and 55.40 ± 17.81%. Contrary to the study on anti-HBV activity of caffeoylquinic acids, there is presently no study that reports the HBV promoting activity of caffeoylquinic acids.

Structure–activity relationship analysis of these caffeoylquinic acids in anti-HBV activity suggested that the presence of caffeoyl groups in the molecule resulted in stronger anti-HBV activity. This conclusion resembles previous study on the structure–activity relationship of these compounds.^34^ The two adjacent phenolic structure and α, β-conjugated unsaturated ester structure of caffeoyl groups increased benzene ring plane conjugation and resulted in the promotion of anti-HBV activity. For simple caffeic acid alone, compounds 6–8, 17 have significantly anti-HBV activity (*p* < 0.001). However, compound 5 expressed inferior anti-HBV activity when compared to the other simple caffeic acid because its relative HBV DNA level (68.35 ± 15.26%) was significantly increased compared to the control group (*p* < 0.001). This is because compound 5 (isovanillic acid, 3-hydroxy-4-methoxybenzoic acid) could not form a benzene ring plane conjugation without a two adjacent phenolic structure, so it exhibited poor activity. For monocaffeoylquinic acid, only compound 12 (3-*O*-caffeoylquinic acid) showed excellent anti-HBV activities (*p* < 0.001). Other isolated monocaffeoylquinic acids, namely 13 (1-*O*-caffeoylquinic acid methyl ester), 14 (4-*O*-caffeoylquinic acid methyl ester), 15 (3-*O*-caffeoylquinic acid methyl ester), and 18 (5-*O*-caffeoylquinic acid methyl ester), did not significantly inhibited the secretion of HBsAg, HBeAg, and HBV DNA replication. These results suggest that carboxyl group in quinic acid was important for the accelerating effect on anti-HBV activities in monocaffeoylquinic acids. However, the caffeoylquinic acid ethyl ester derivatives (9) showed the best anti-HBV activity (*p* < 0.001). Perhaps further study on the structure–activity relationship of monocaffeoylquinic acids is worth discussing. For dicaffeoylquinic acids, compared to their structural analog (20), new compounds 1 and 2 have only one caffeoyl group in the molecule, so they exhibited weaker activities than that of compound 20. Moreover, the anti-HBV activity of new compound 3 was far better than that of compound 20, which could be due to the *cis*-caffeoyl group in compound 3. The above results may suggest that the existence and configuration of the double bond moiety in caffeoyl group is important for anti-HBV activity. Tricaffeoylquinic acid, compound 23 (3,4,5-tri-*O*-caffeoylquinic acid methyl ester), had a steric hindrance because it contained three caffeoyl groups. The increased steric hindrance effect inhibited the anti-HBV activity of compound 23. Unfortunately, the relative HBsAg level (172.39 ± 2.59%) and HBV DNA level (55.40 ± 17.81%) of compound 23 was significantly promoted (*p* < 0.001). Therefore, more studies are necessary to further understand the anti-HBV action mechanism of caffeoylquinic acid derivatives.

## Conclusion

This study focuses on seeking anti-HBV agents from *L. japonica* flower buds, which are widely used in traditional Chinese medicine, healthy food, and cosmetics. Four new caffeoylquinic acids (1–4), five simple caffeic acids (5–8, 17) and fourteen known caffeoylquinic acids (9–16, 18–23) were isolated from *L. japonica*, of which caffeoylquinic acids exhibited potent HBV inhibitory activities. The results indicated that the HBV inhibitory activity of *L. japonica* flower buds may occur primarily because of the amount of caffeoylquinic acids present and this could serve as anti-HBV agents for functional food or medical use. Also, we found that compound 23 appeared to significantly promote HBsAg and HBeAg secretion, and HBV DNA replication. Unlike the study on anti-HBV activity of caffeoylquinic acids, there is no current manuscript that reports caffeoylquinic acids can promote HBV activity.

## Conflicts of interest

The authors declare no competing financial interest.

## Supplementary Material

RA-008-C8RA07549B-s001
