# Supplementary material for: Novel caffeoylquinic acid derivatives from Lonicera japonica Thunb. flower buds exert pronounced anti-HBV activities
Source: RSC Adv. 2018 Oct 15;8(62):35374–85. doi: 10.1039/c8ra07549b (PMC9088017; doi:10.1039/c8ra07549b)
Supplement: RA-008-C8RA07549B-s001 [file RA-008-C8RA07549B-s001.pdf]

Electronic Supplementary Material (ESI) for RSC Advances.
This journal is © The Royal Society of Chemistry 2018

Novel Caffeoylquinic Acid Derivatives from *Lonicera japonica* Flower Buds Exert Pronounced Anti-HBV Activities

Lanlan Ge [abc‡], Haoqiang Wan [ad‡], Shuming Tang [e], Haixia Chen [e], Jiemei Li [ad], Keda Zhang [a], Boping Zhou [a], Jia Fei [c], Shiping Wu [b*], Xiaobin Zeng [ad*]

[a]*Center Lab of Longhua Branch, Shenzhen People's Hospital, 2nd Clinical Medical College of Jinan University, Shenzhen 518120, Guangdong Province, China. E-mail: zengxiaobin1983@163.com; Fax: +86-755-28100877; Tel: +86-755-27745118.*

[b]*Department of Infectious disease, Shenzhen People's Hospital, 2nd Clinical Medical College of Jinan University, Shenzhen 518120, Guangdong Province, China. E-mail: wupoem@126.com*

[c]*Integrated Chinese and Western Medicine Postdoctoral research station, Jinan University, Guangzhou 510632, Guangdong Province, China*

[d]*Department of Pathology (Longhua Branch), Shenzhen People's Hospital, 2nd Clinical Medical College of Jinan University, Shenzhen 518120, Guangdong Province, China*

[e]*Laboratory Department of Longhua Branch, Shenzhen People's Hospital, 2nd Clinical Medical College of Jinan University, Shenzhen 518120, Guangdong Province, China*

[‡] These authors have contributed equally to this work

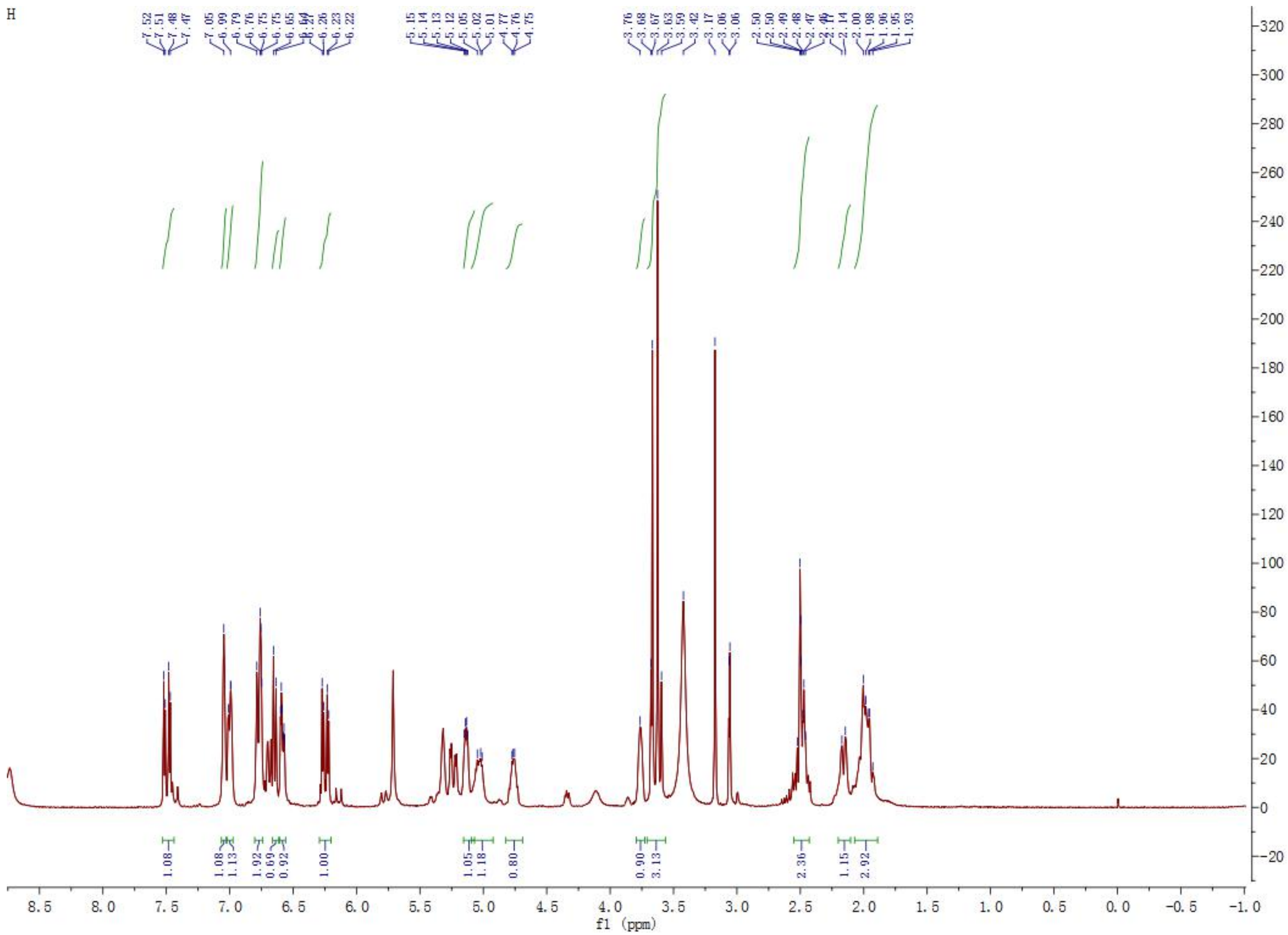

^{1}H-NMR spectrum of compound **1**

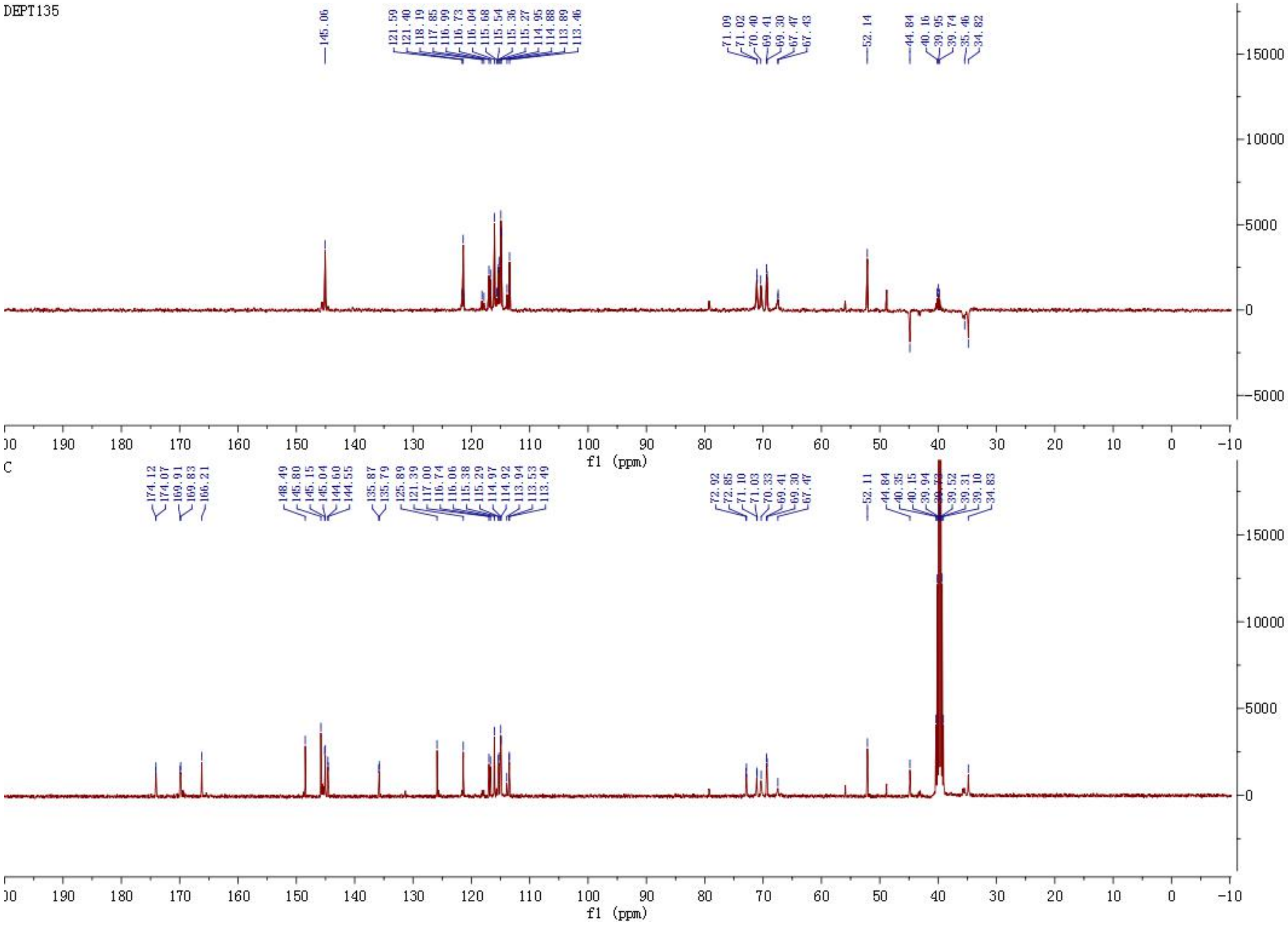

^{13}C-NMR spectrum of compound **1**

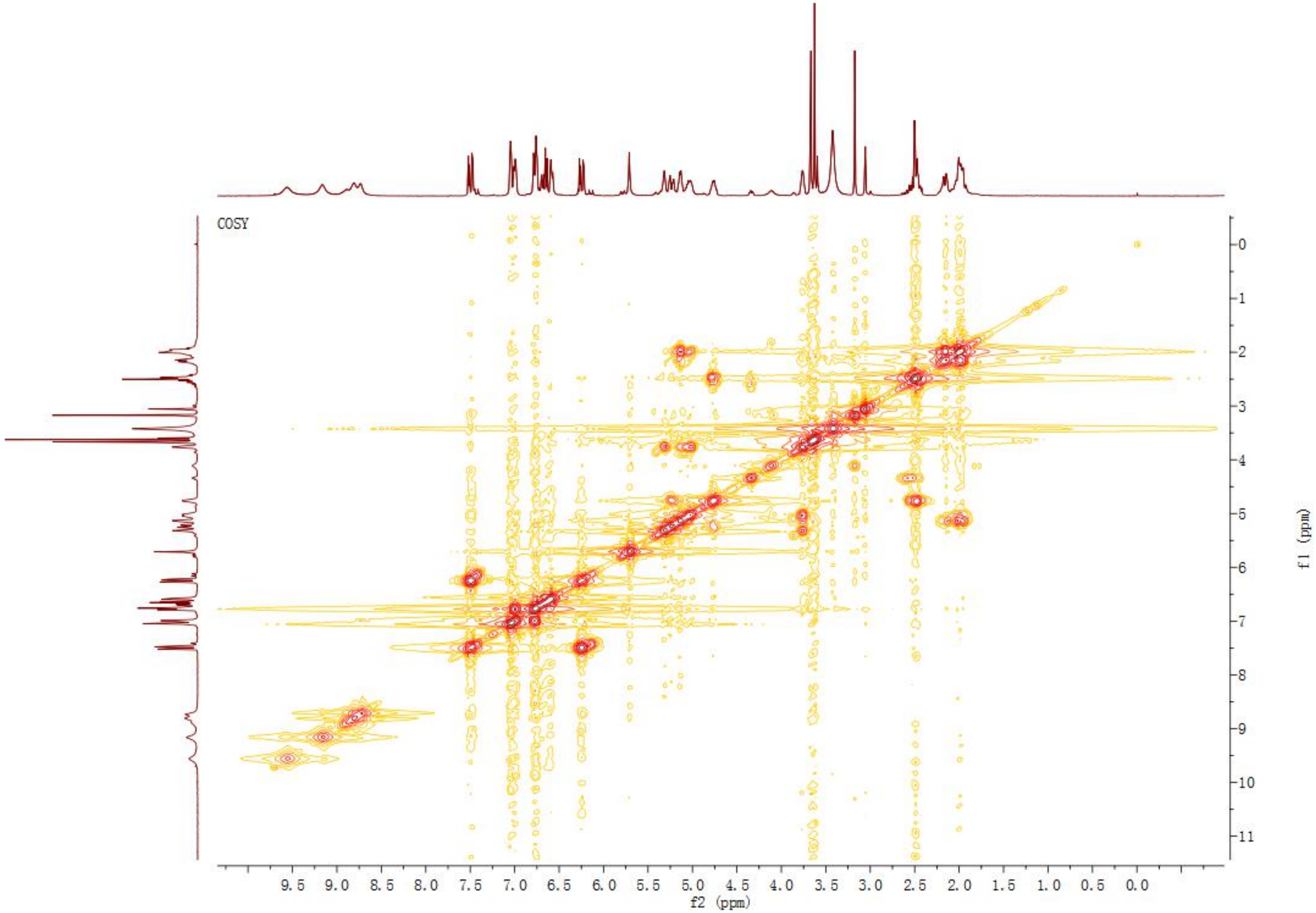

^1H-^1H COSY spectrum of compound **1**

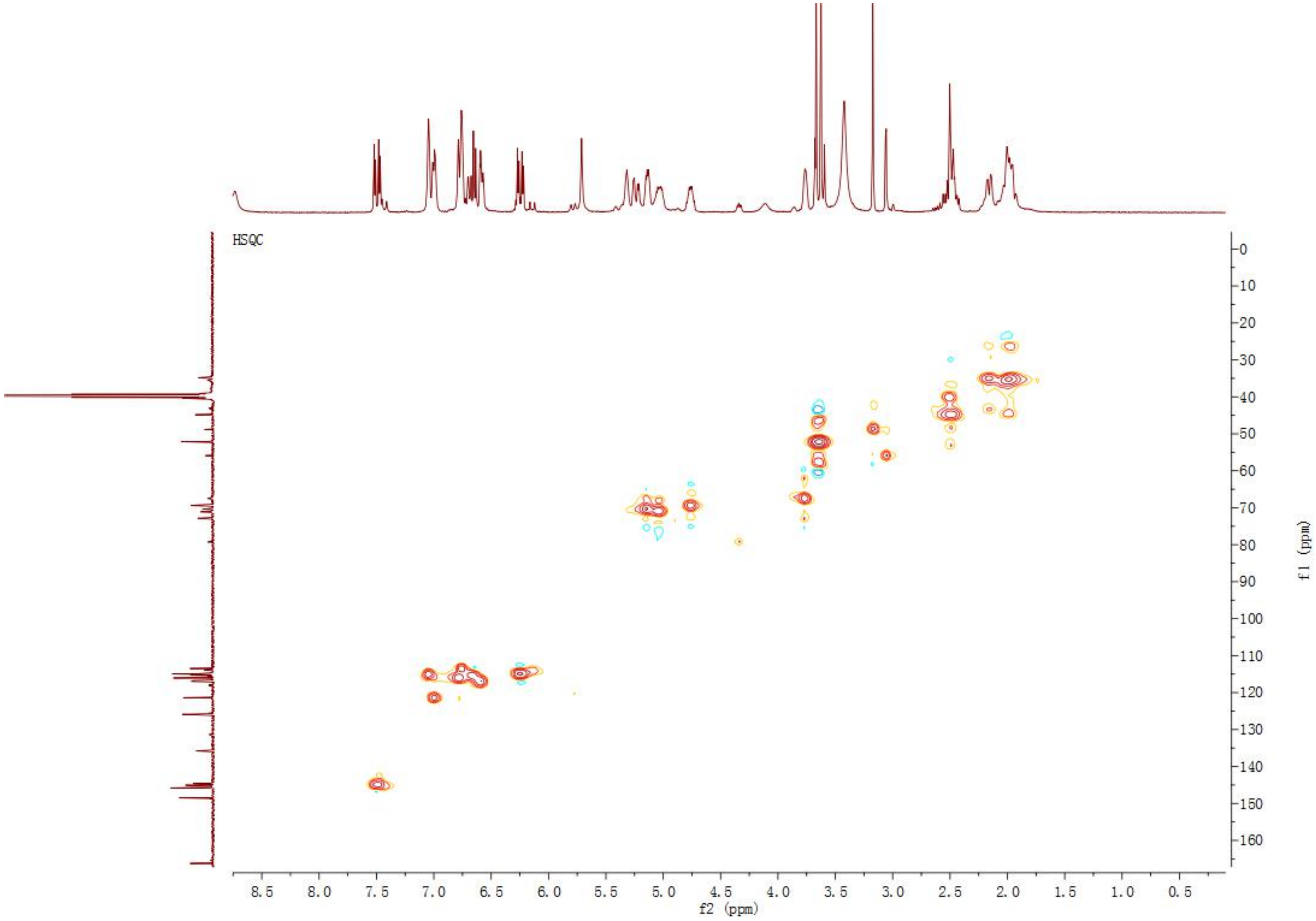

HSQC spectrum of compound **1**

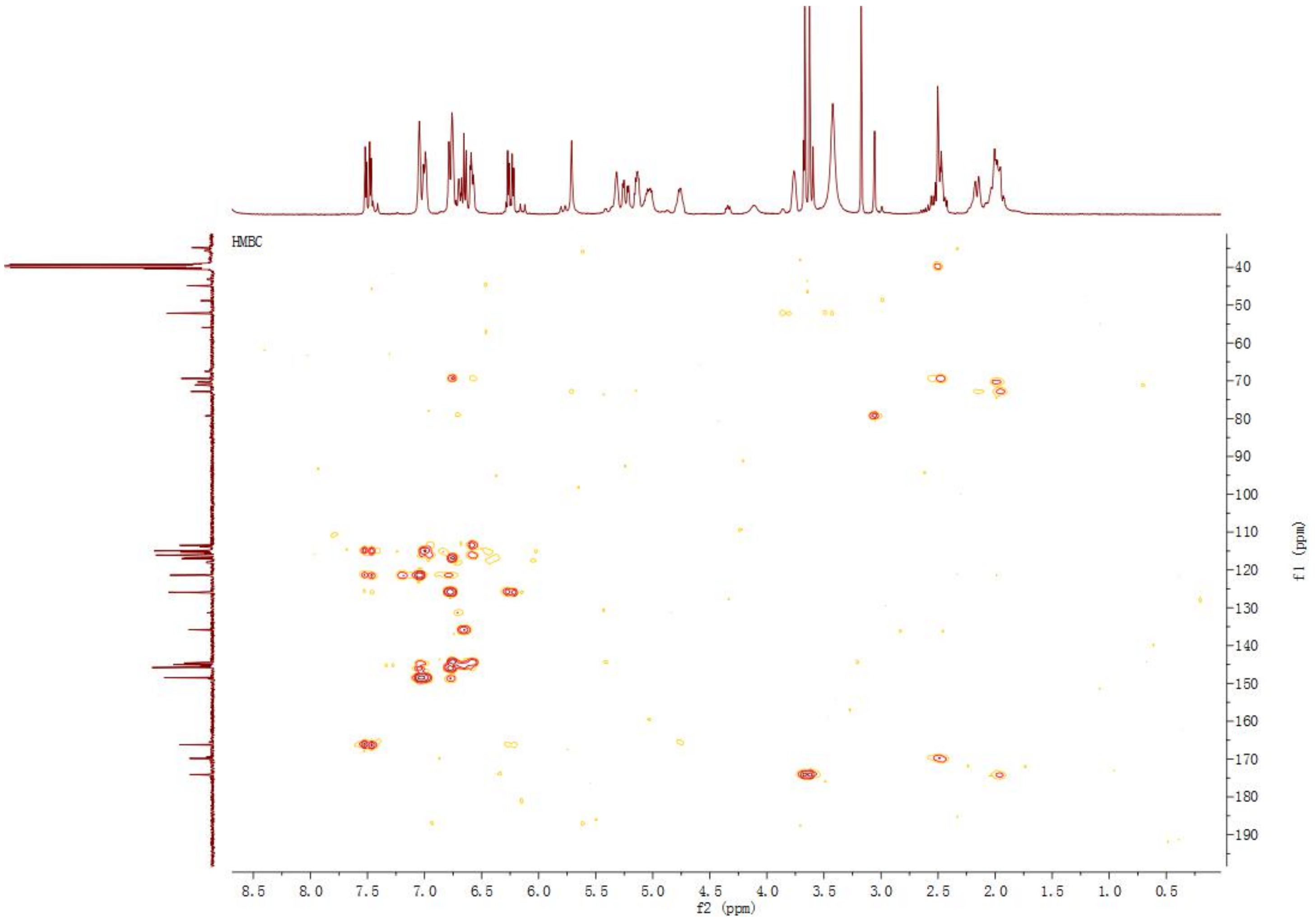

HMBC spectrum of compound **1**

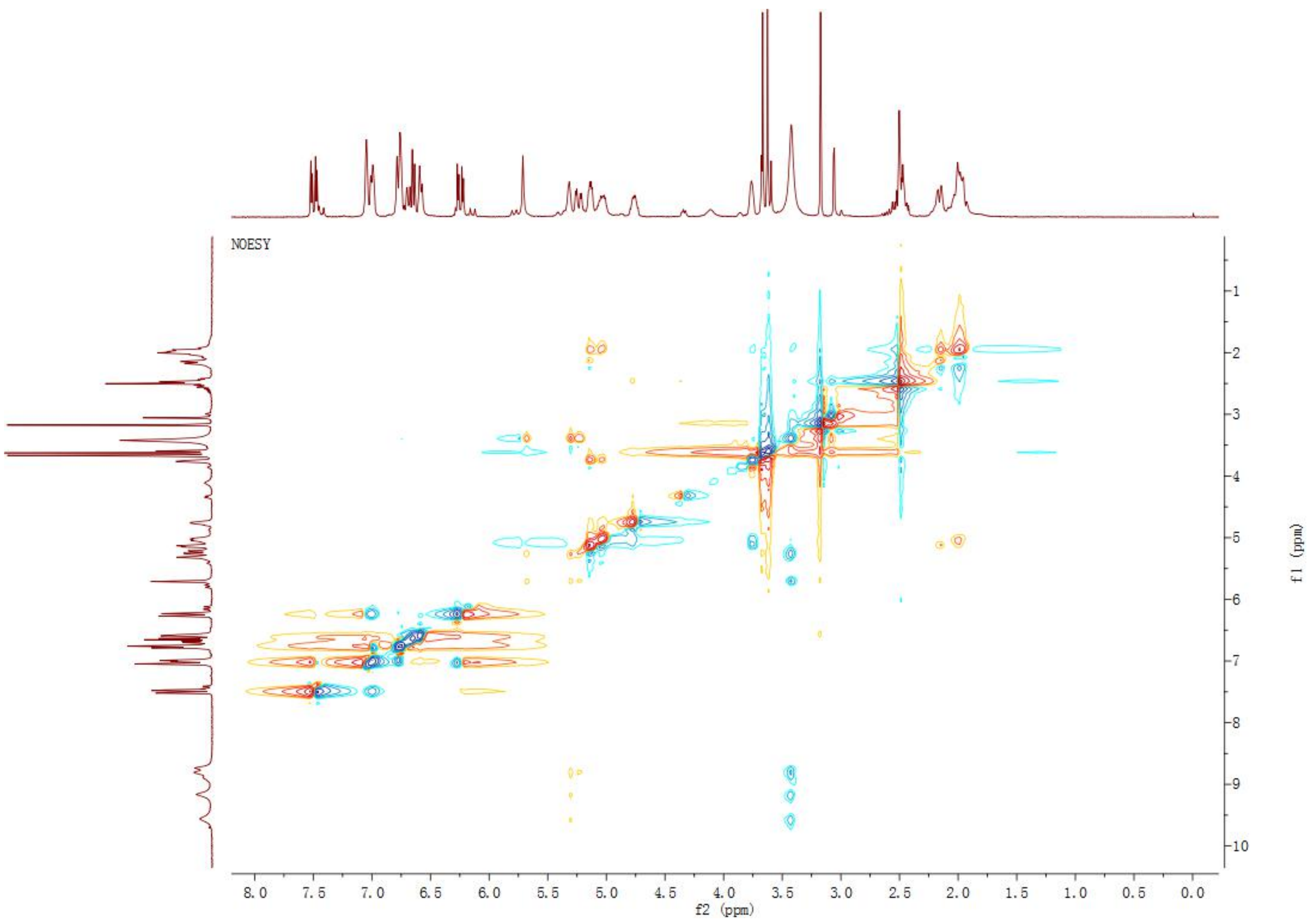

NOESY spectrum of compound **1**

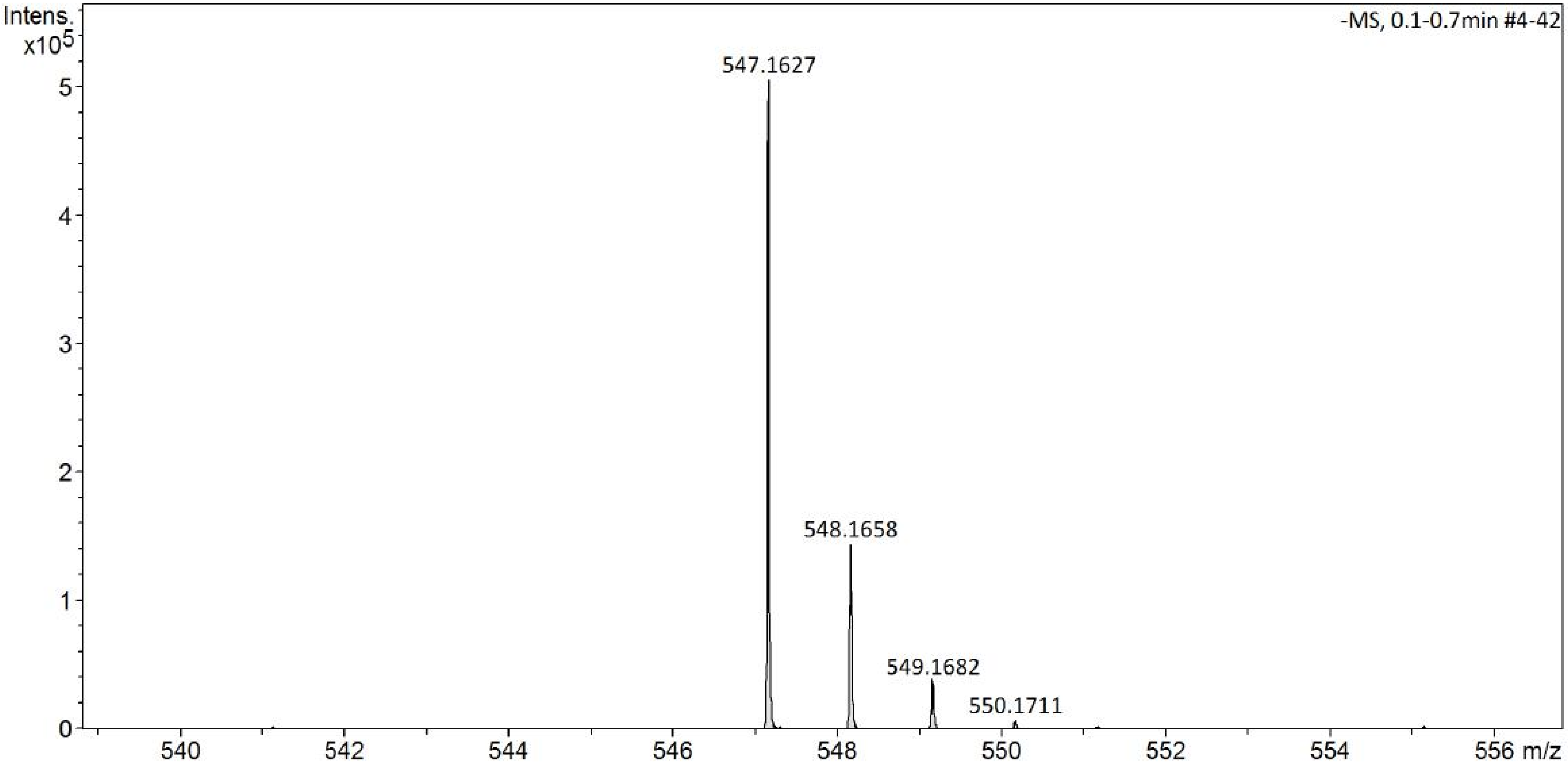

HR-ESIMS spectrum of compound **1**

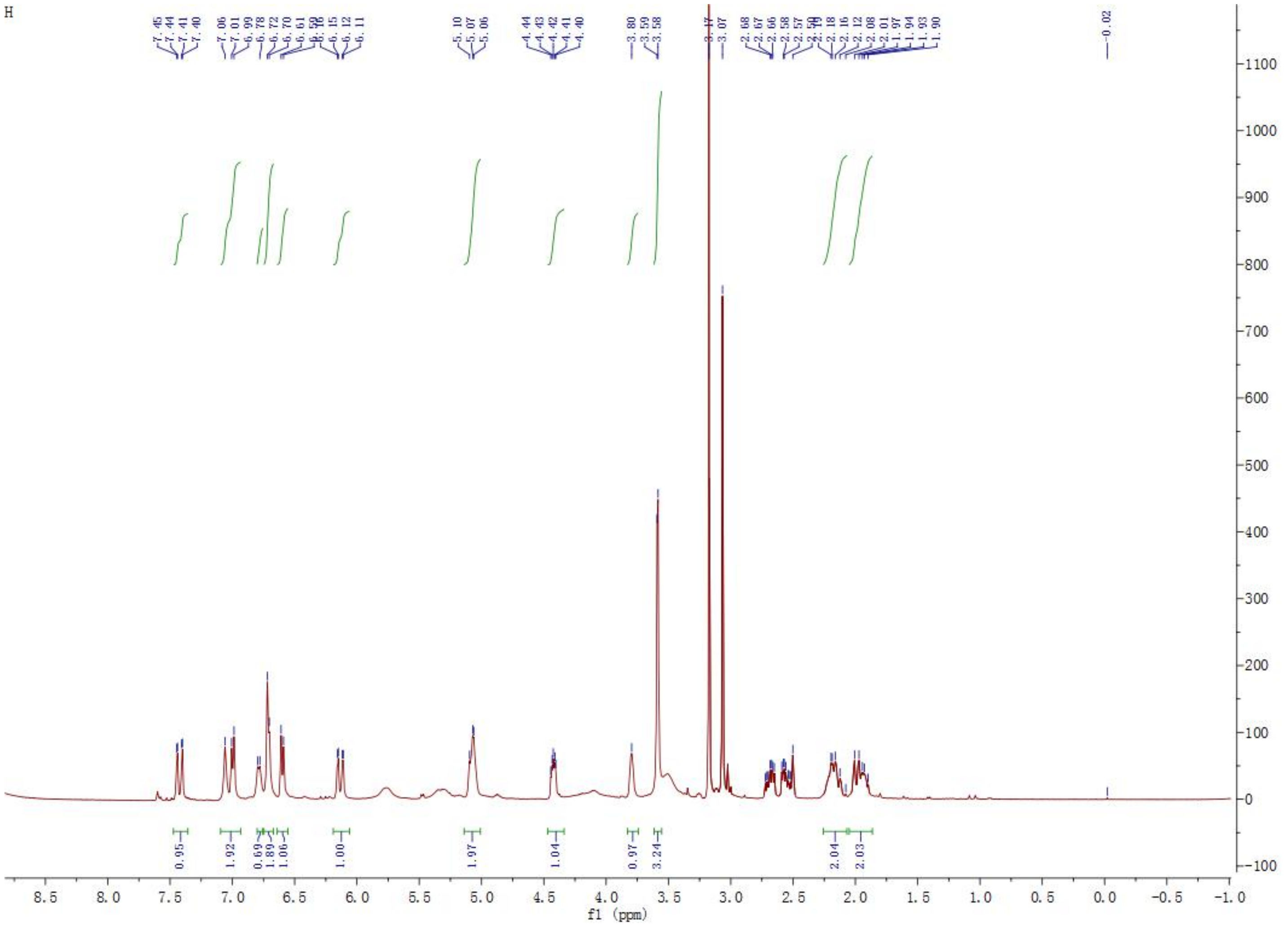

^{1}H-NMR spectrum of compound **2**

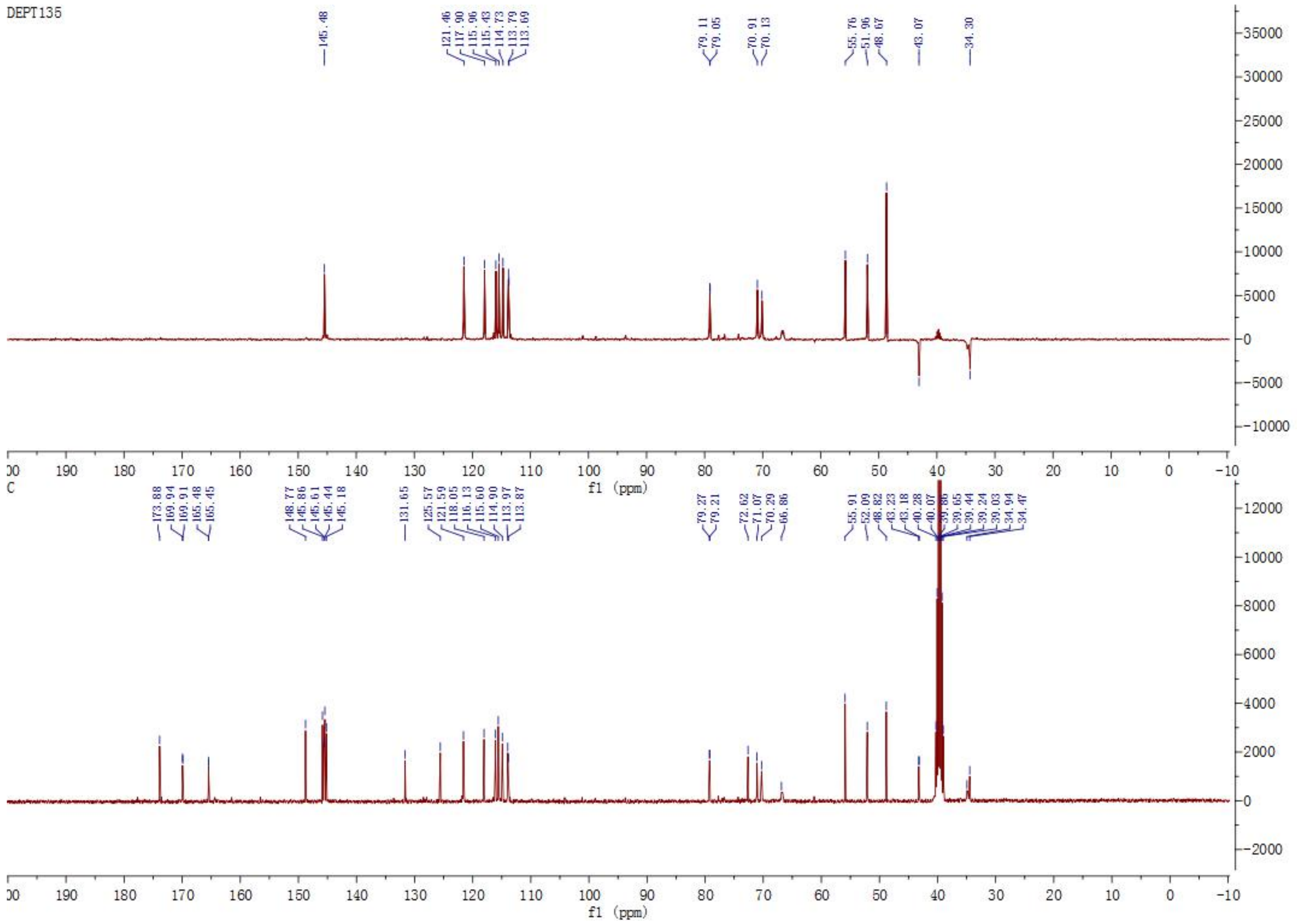

^{13}C-NMR spectrum of compound **2**

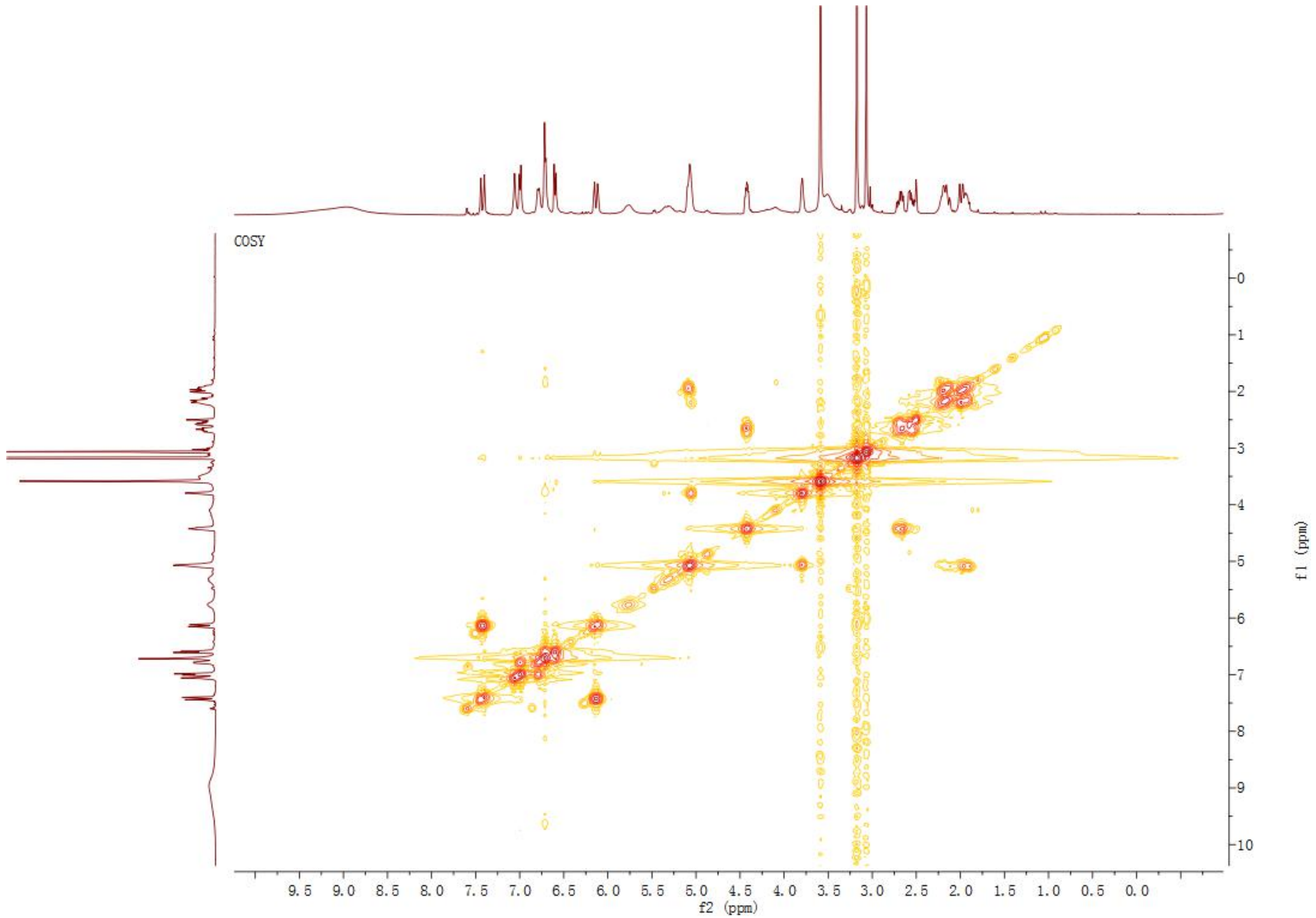

^{1}H-^{1}H COSY spectrum of compound **2**

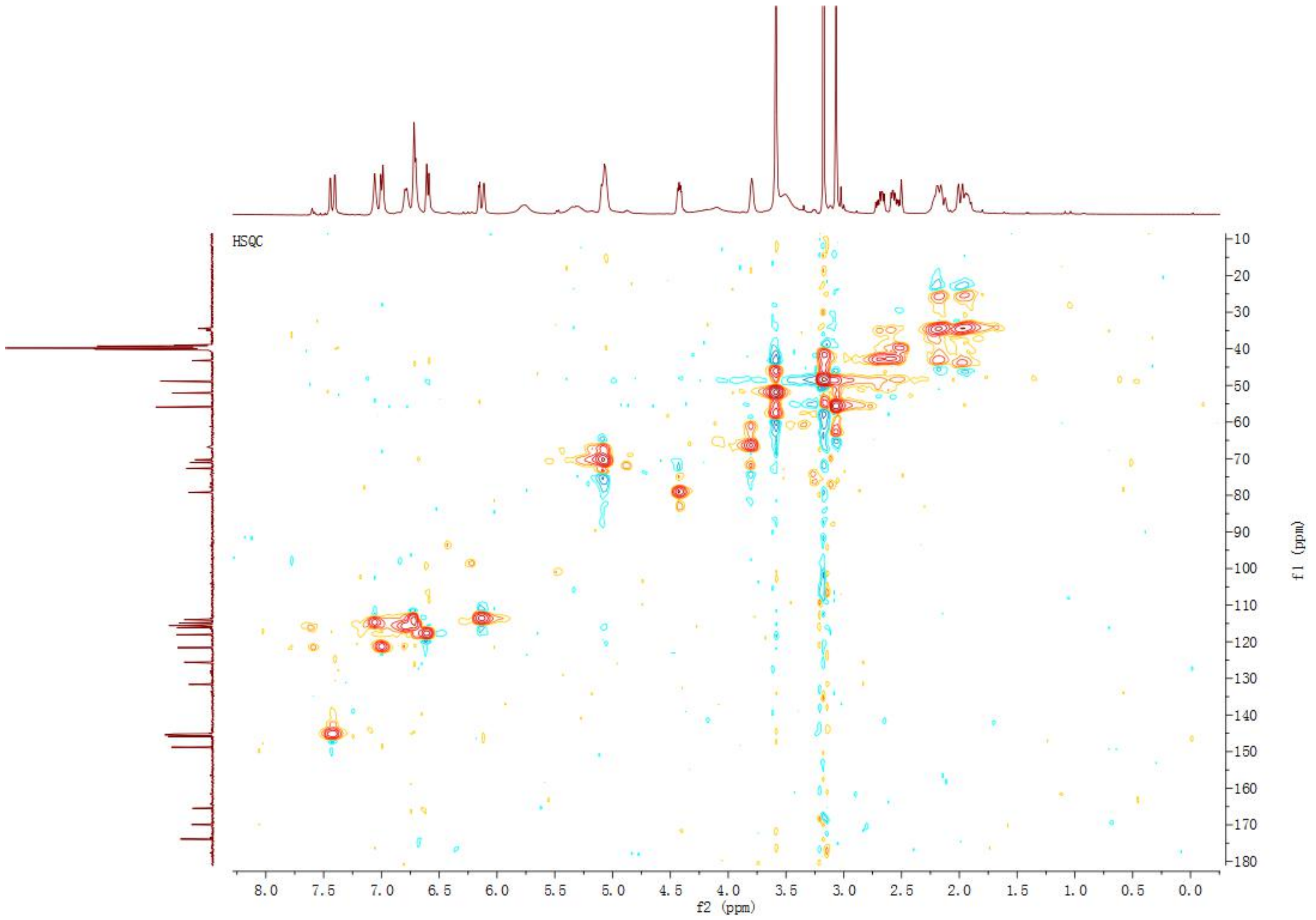

HSQC spectrum of compound **2**

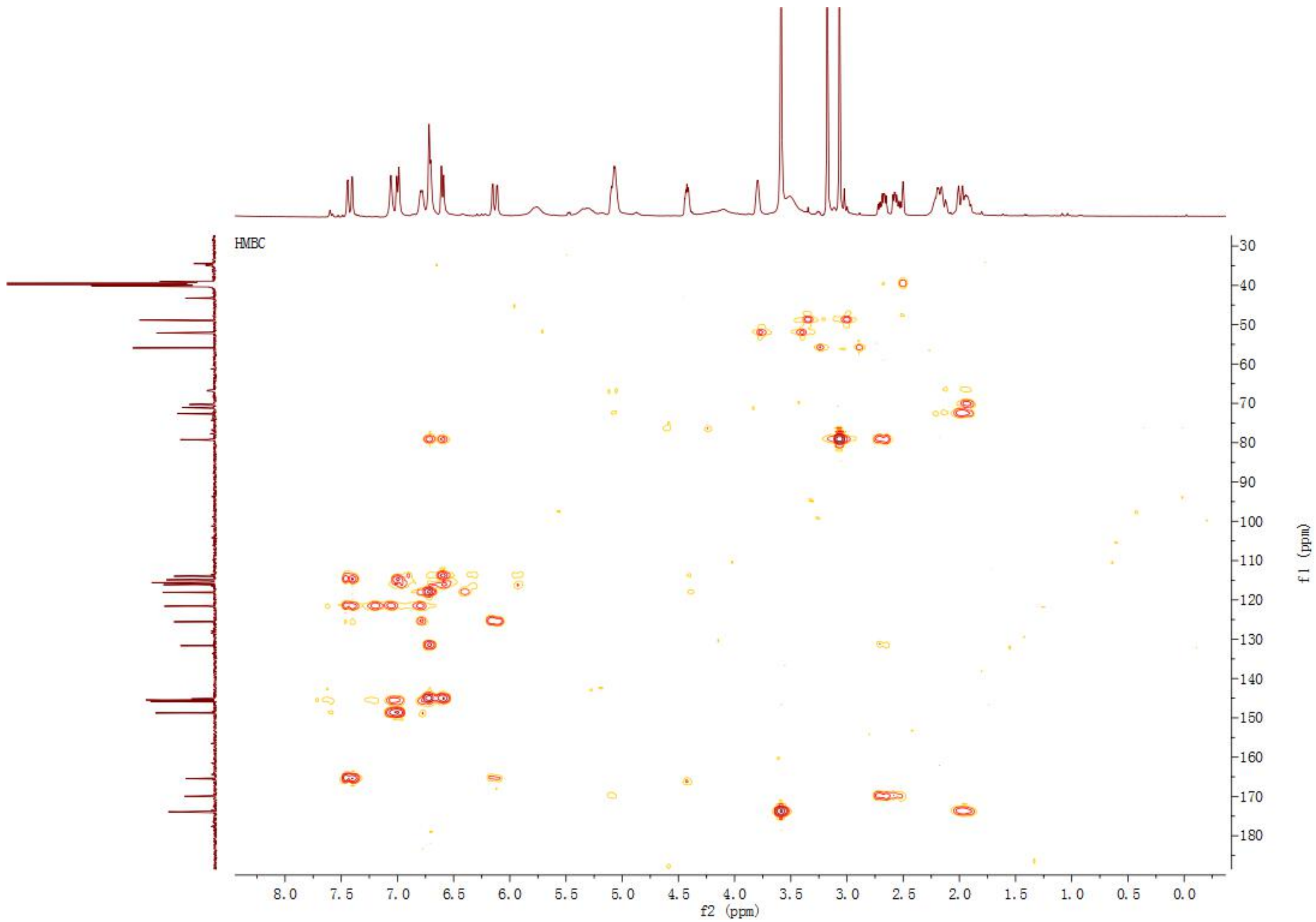

HMBC spectrum of compound **2**

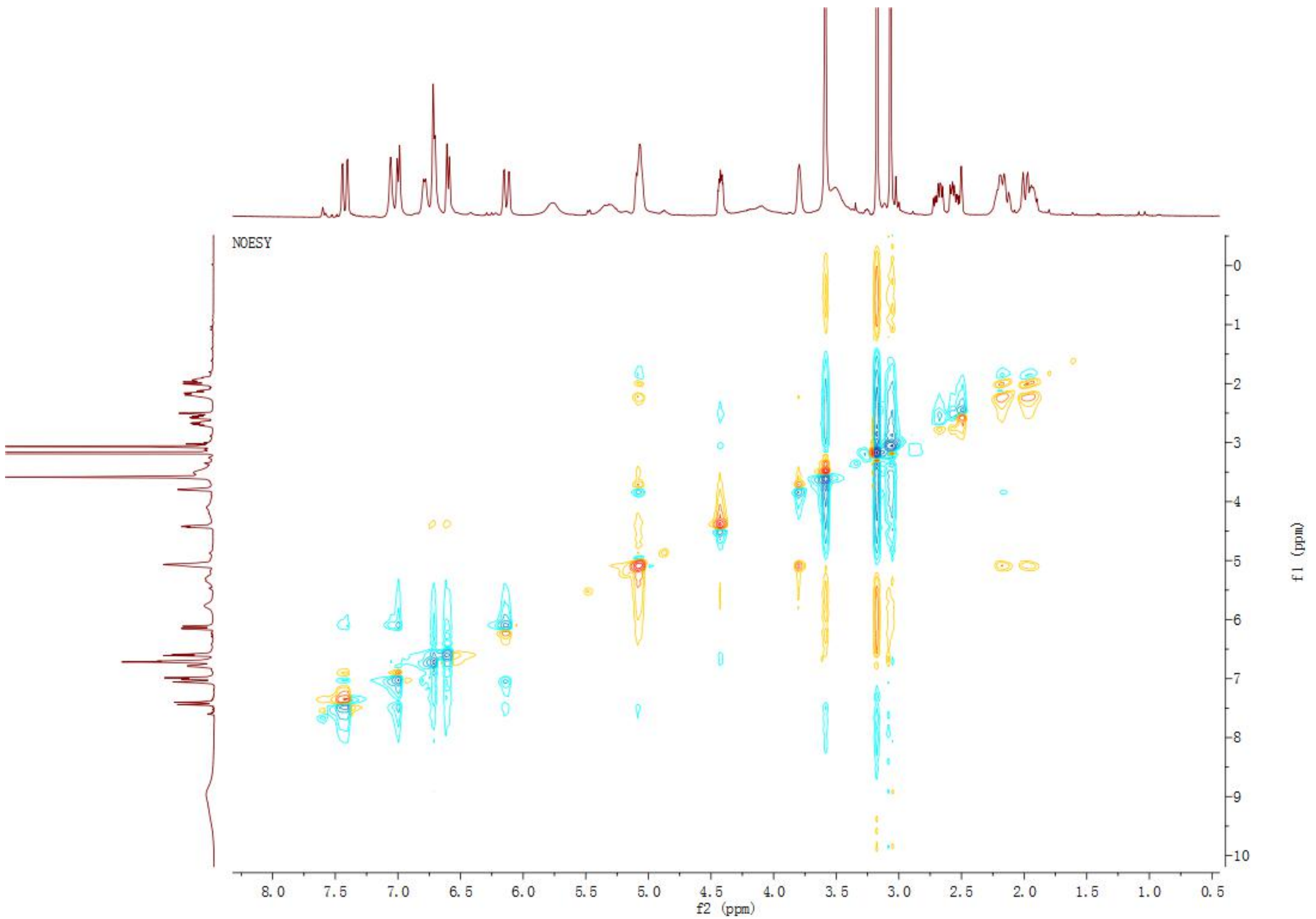

NOESY spectrum of compound **2**

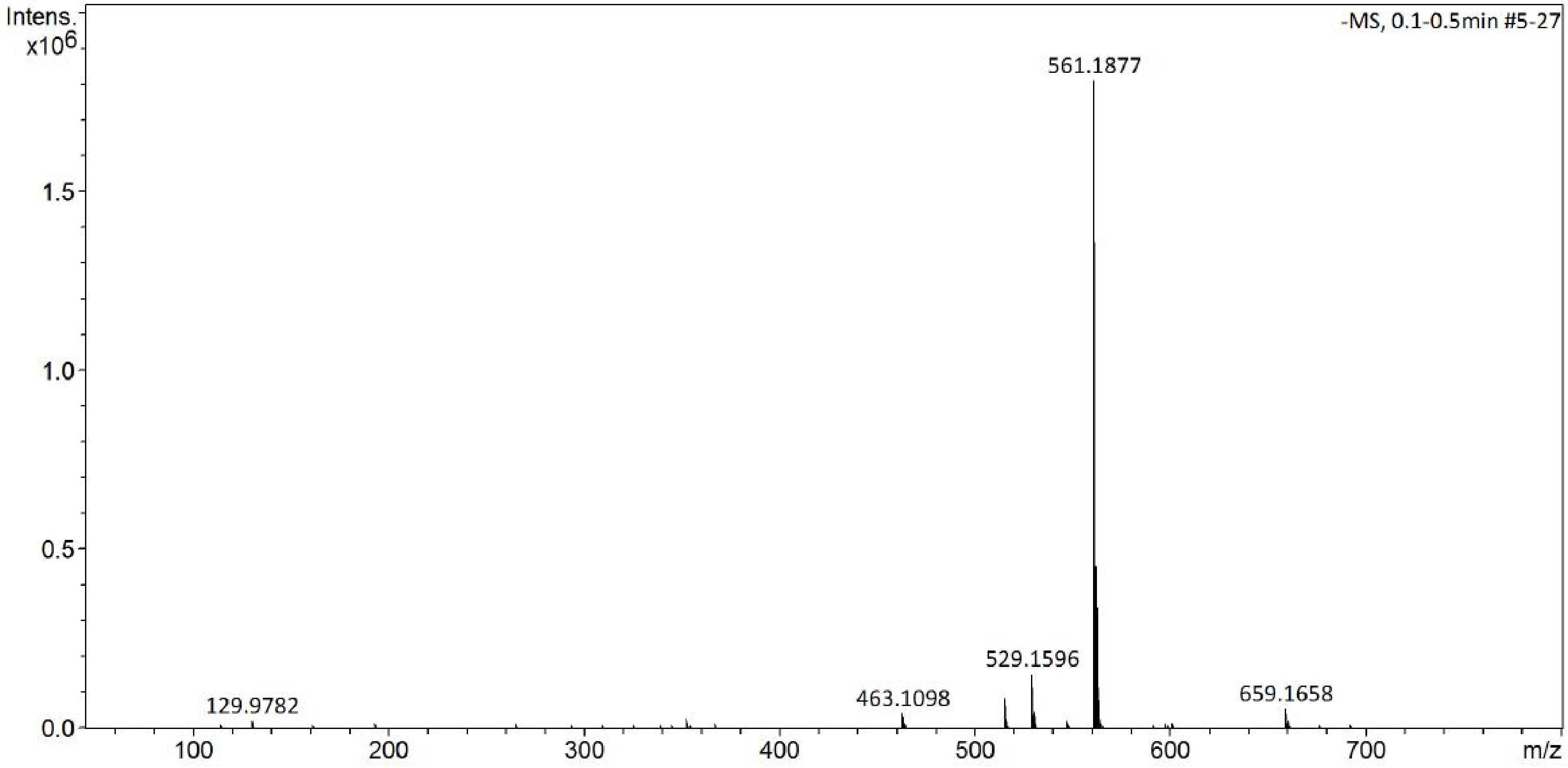

HR-ESIMS spectrum of compound **2**

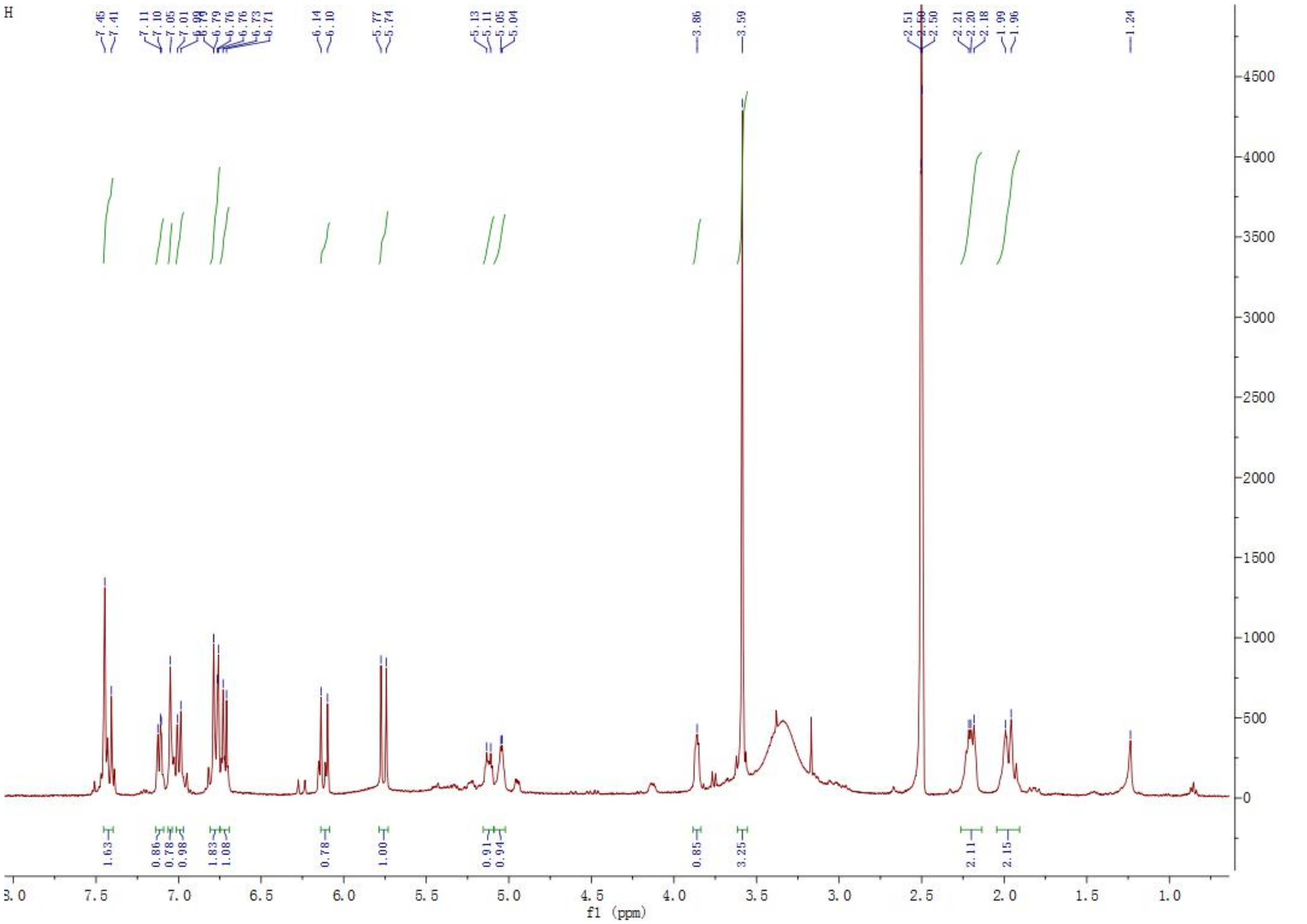

^{1}H-NMR spectrum of compound **3**

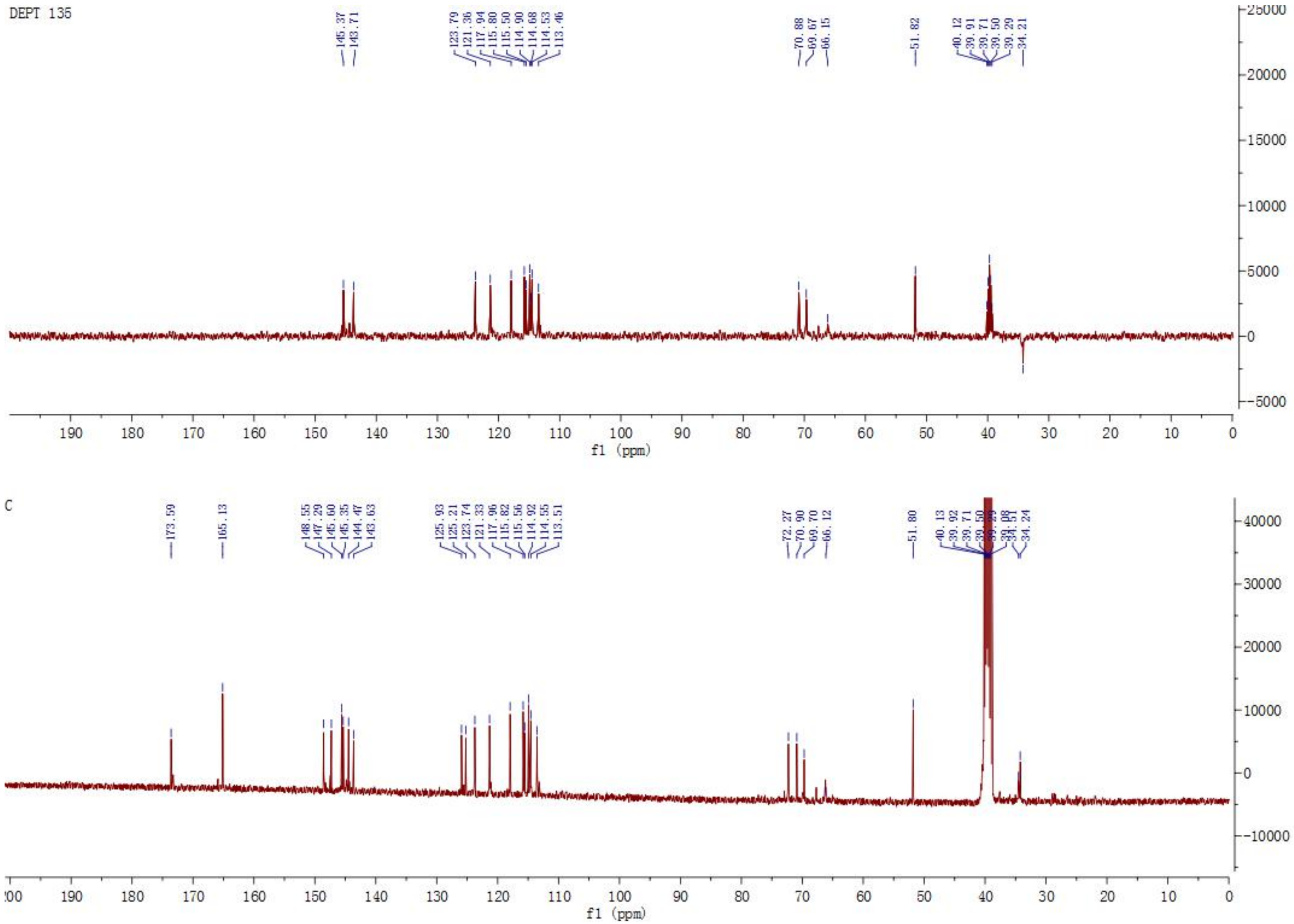

^{13}C-NMR spectrum of compound **3**

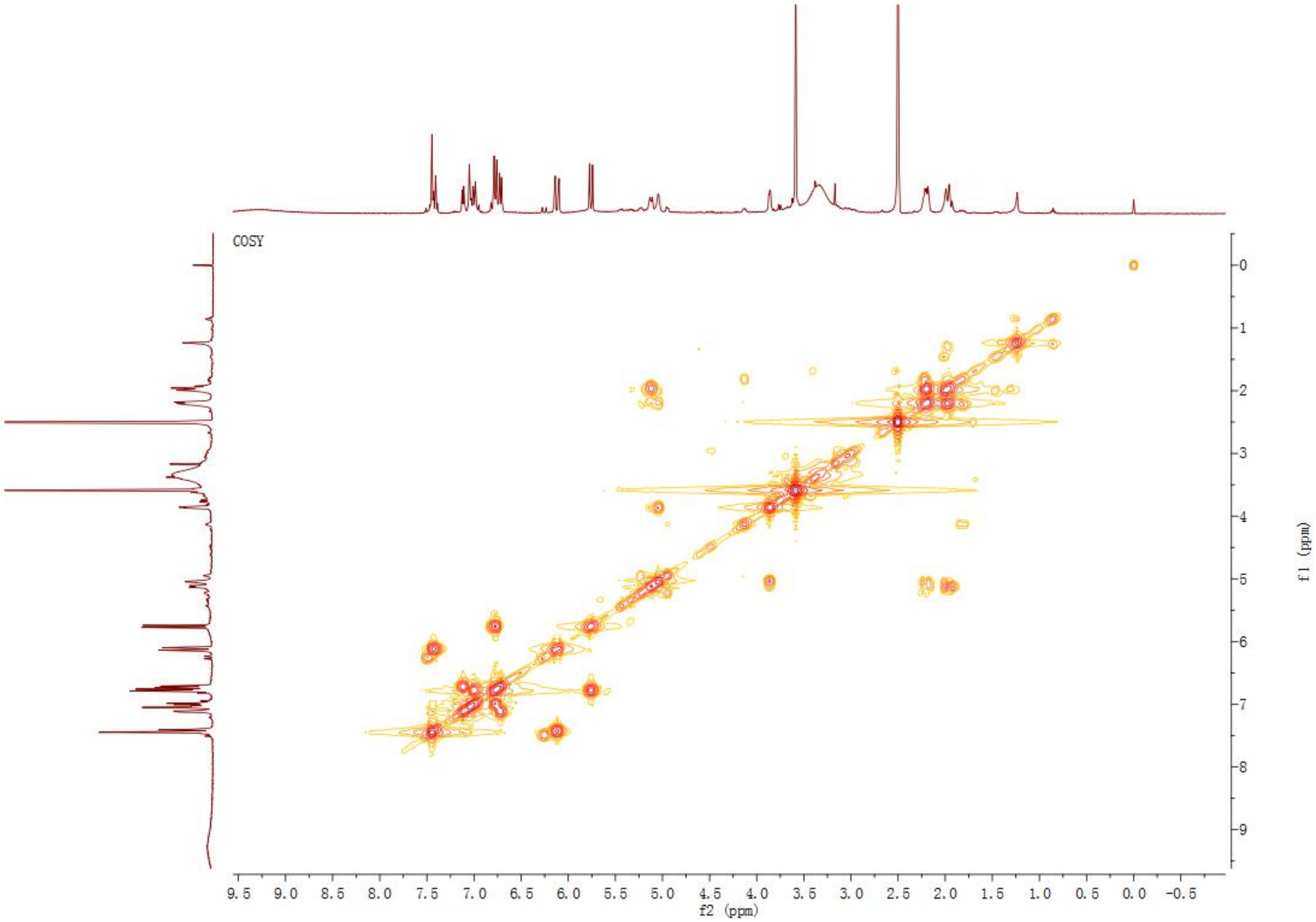

^1H-^1H COSY spectrum of compound **3**

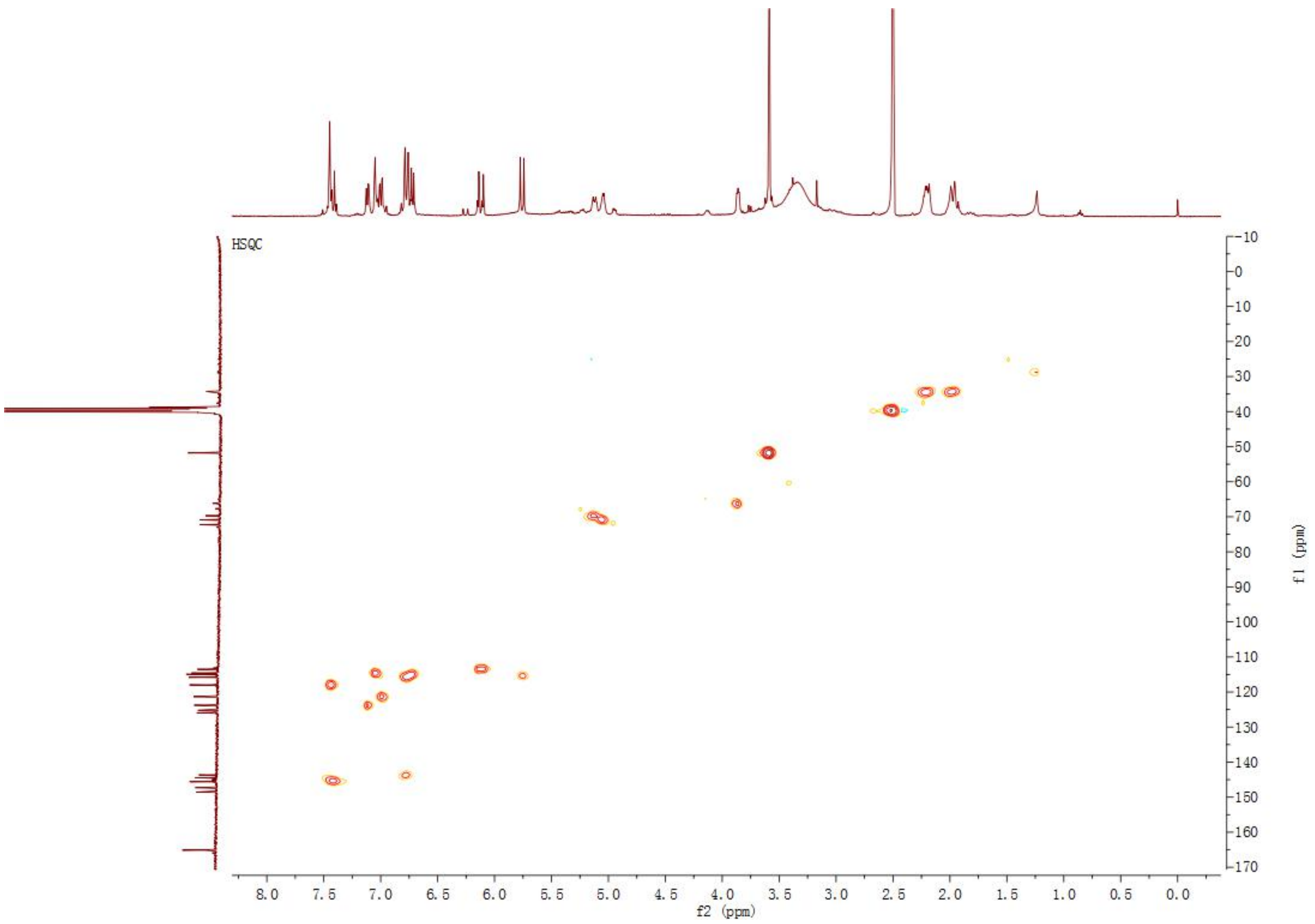

HSQC spectrum of compound **3**

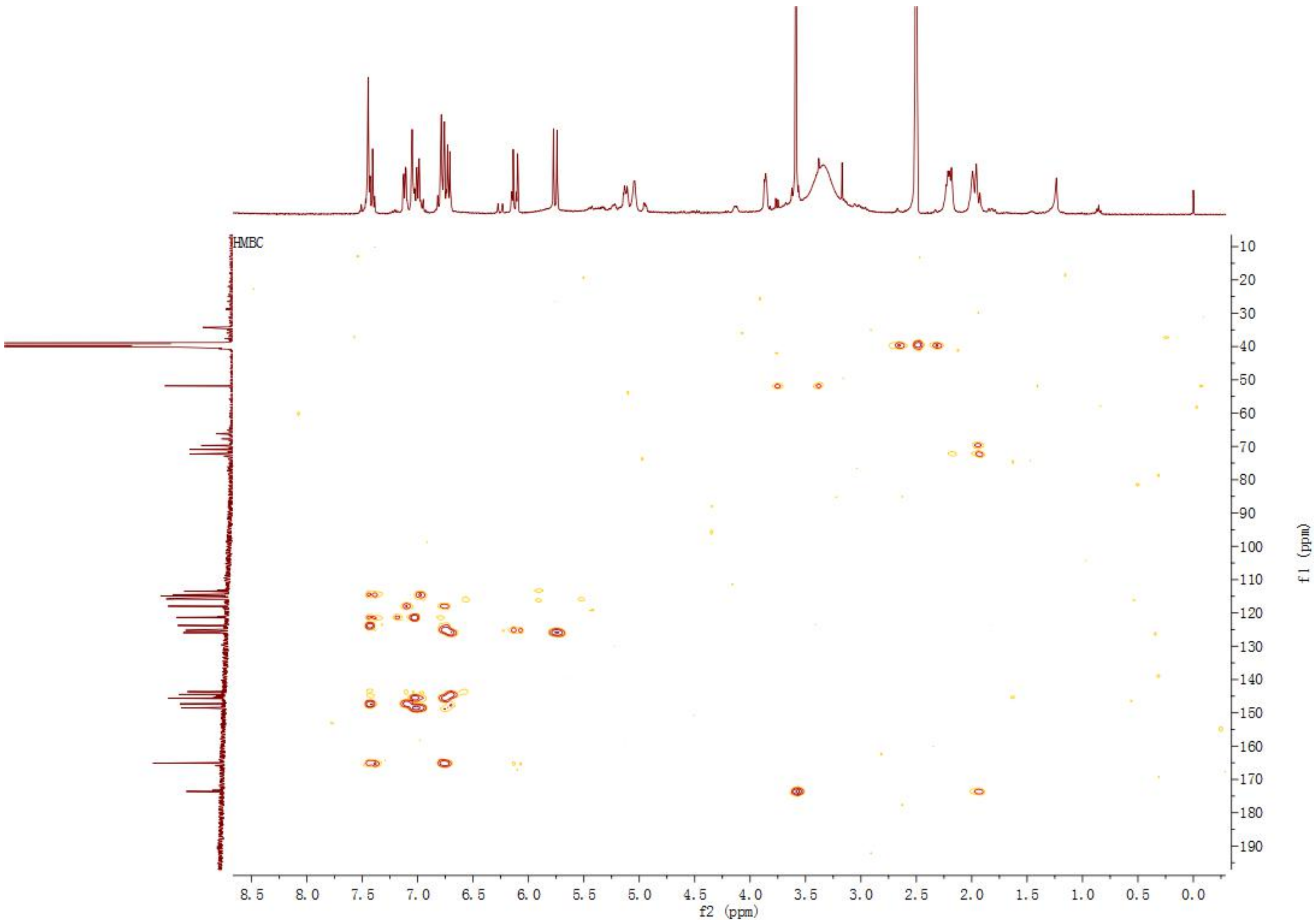

HMBC spectrum of compound **3**

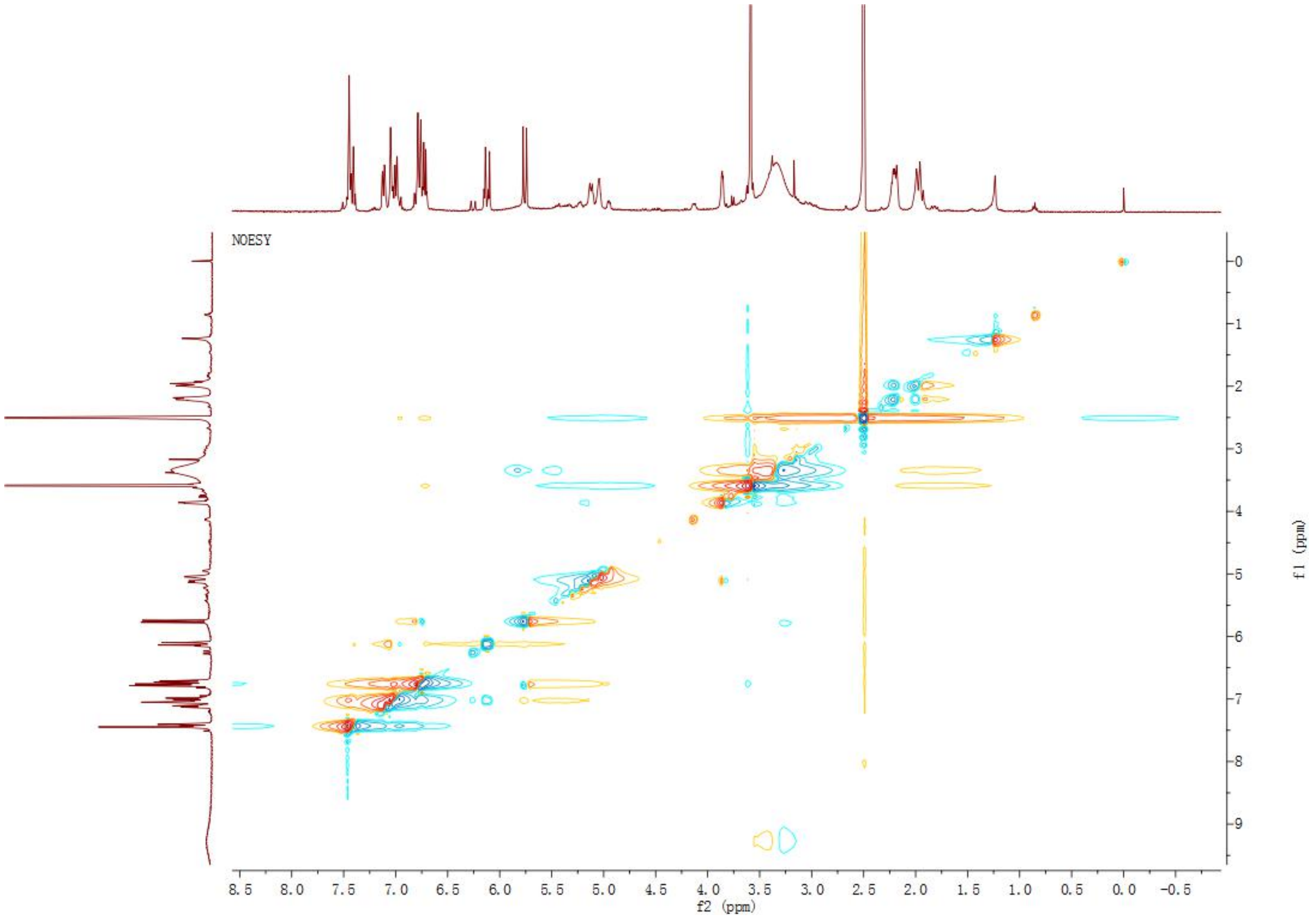

NOESY spectrum of compound **3**

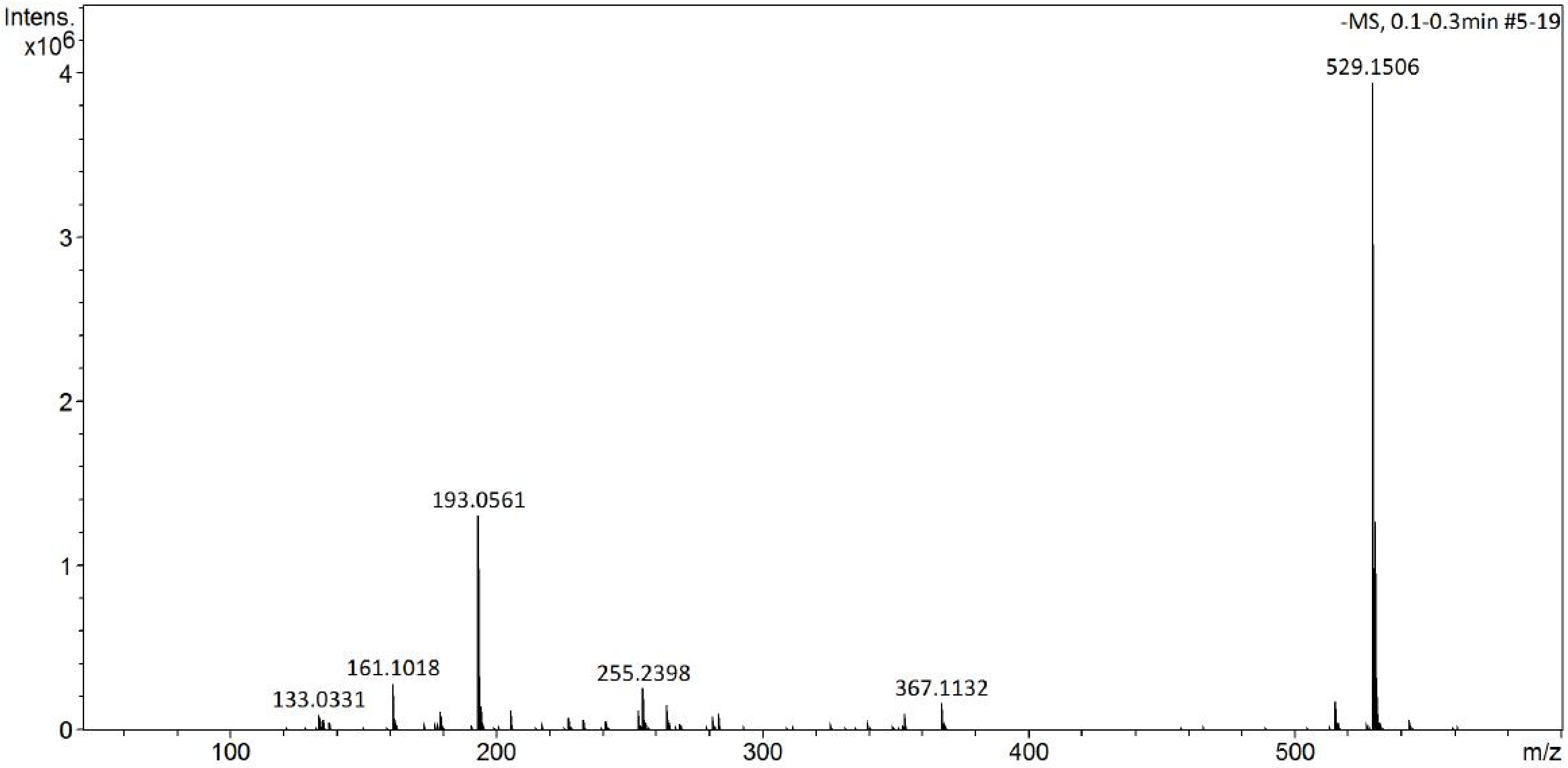

HR-ESIMS spectrum of compound **3**

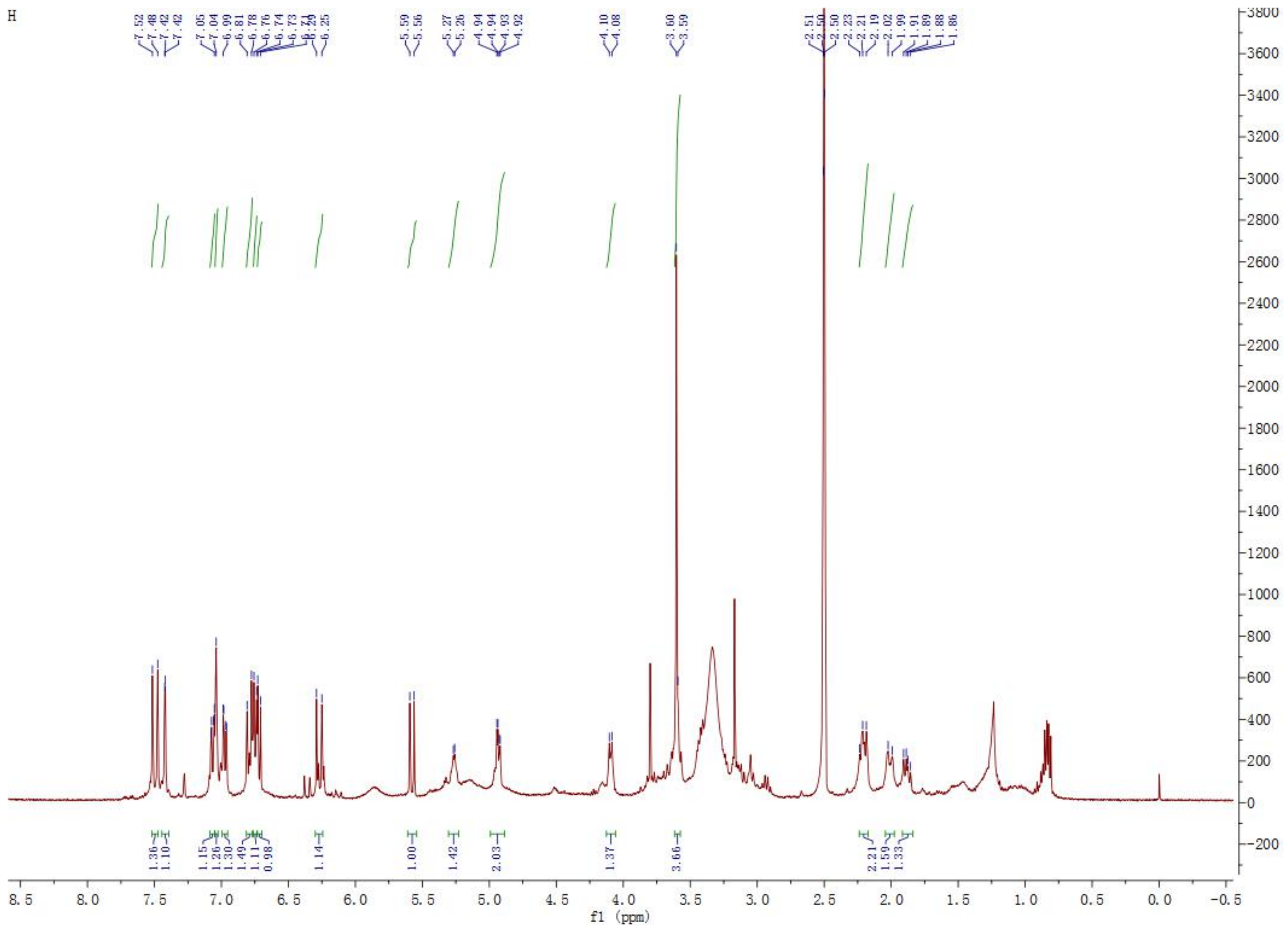

^{1}H-NMR spectrum of compound **4**

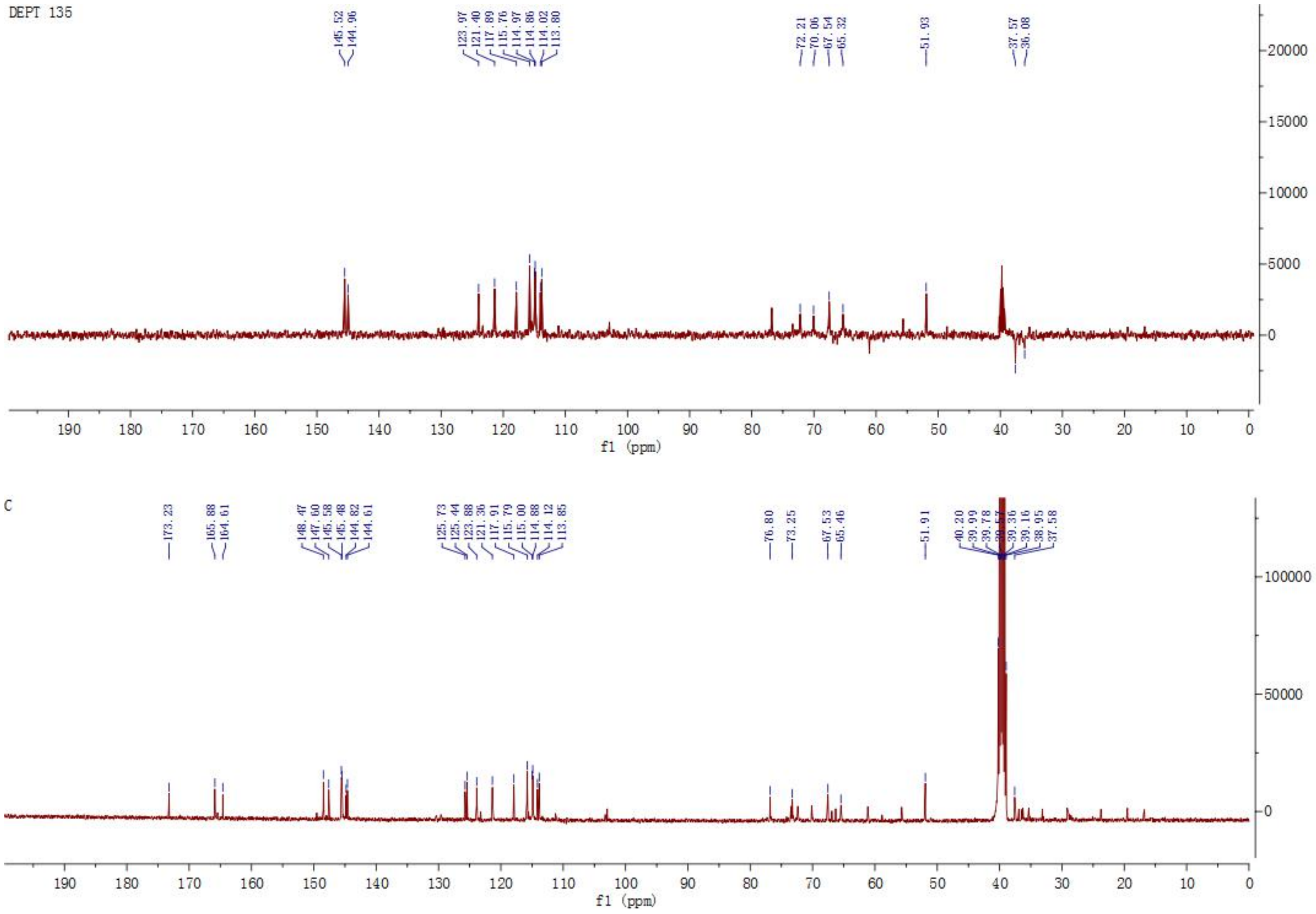

^{13}C-NMR spectrum of compound **4**

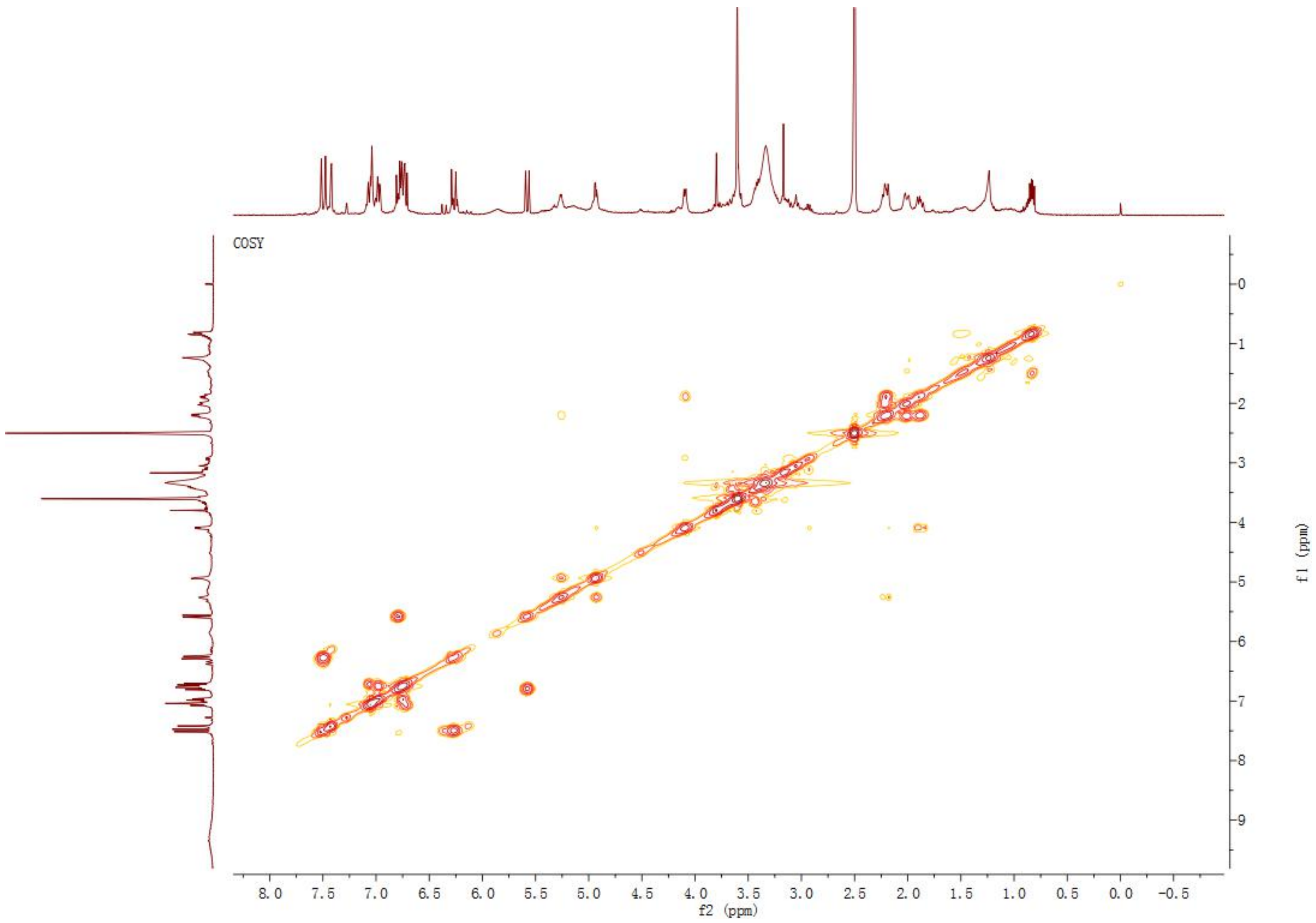

^{1}H-^{1}H COSY spectrum of compound **4**

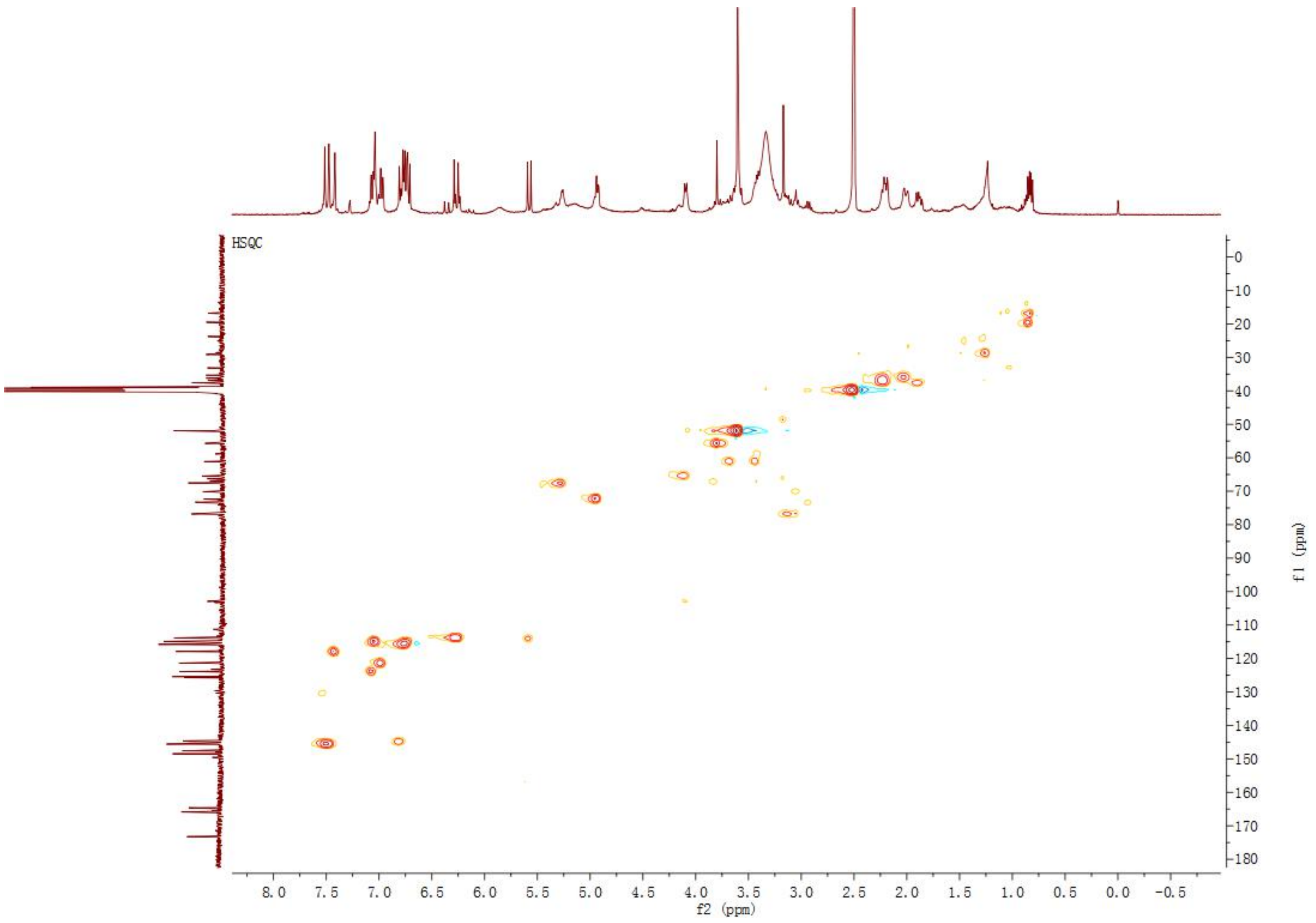

HSQC spectrum of compound **4**

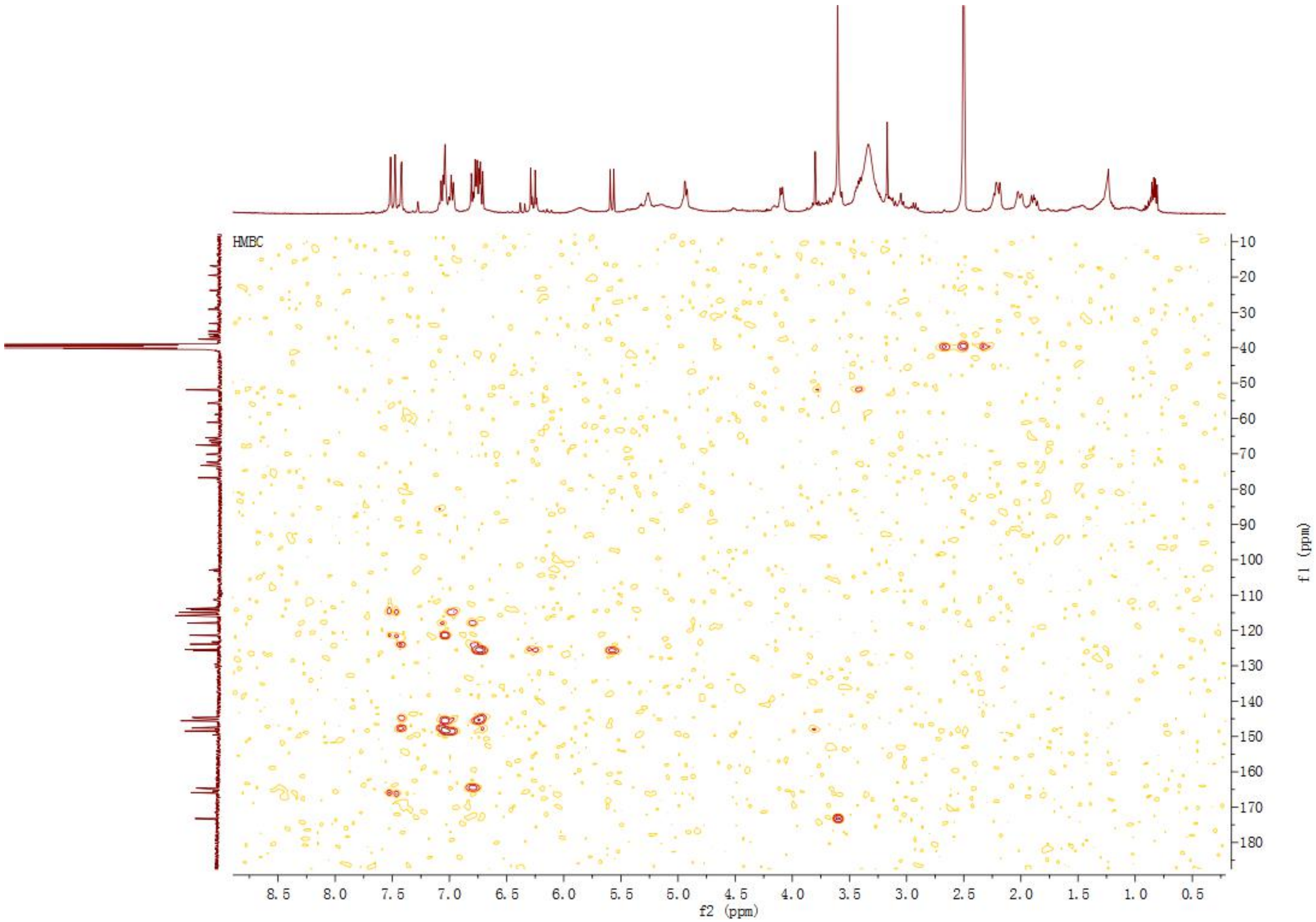

HMBC spectrum of compound **4**

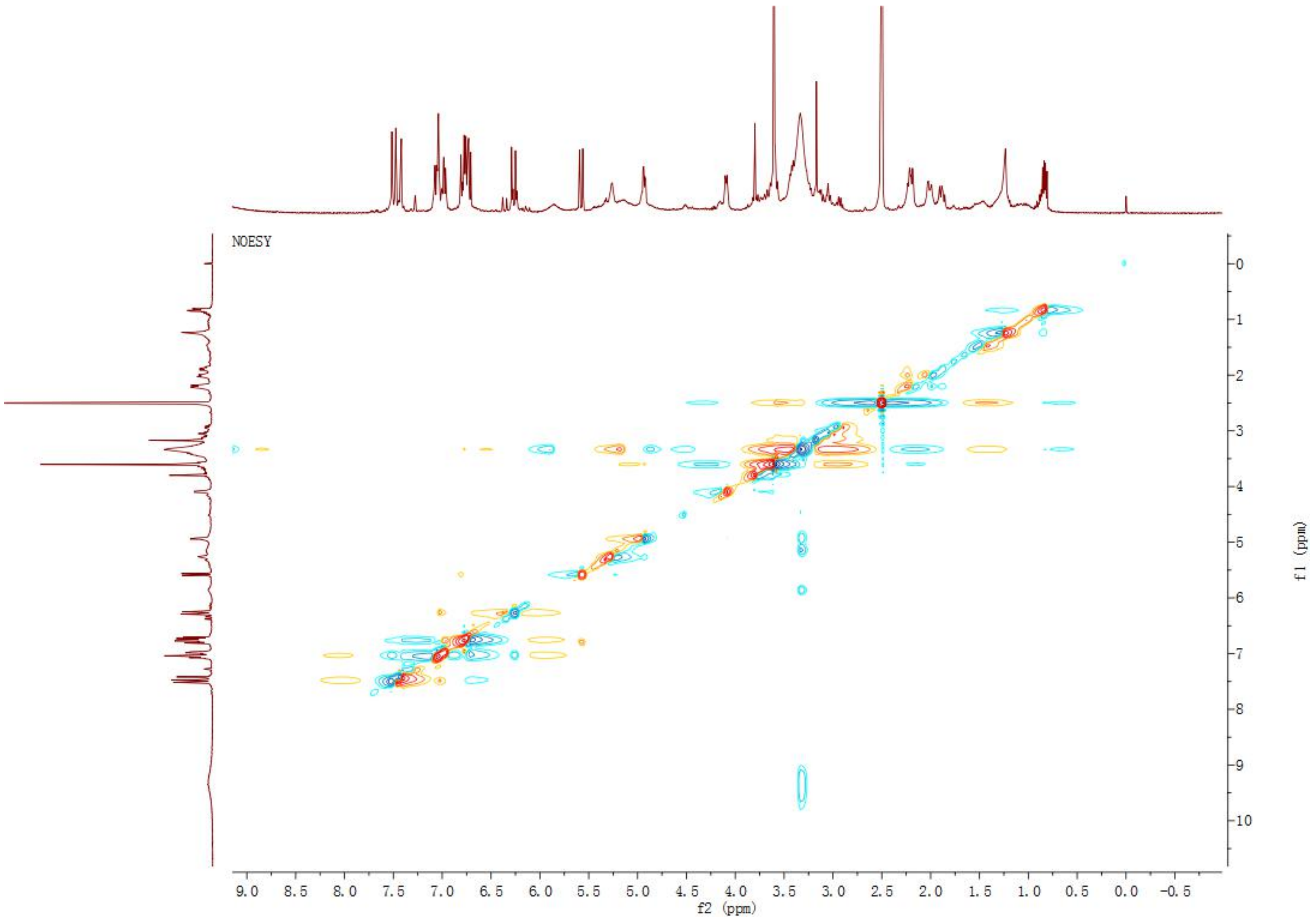

NOESY spectrum of compound **4**

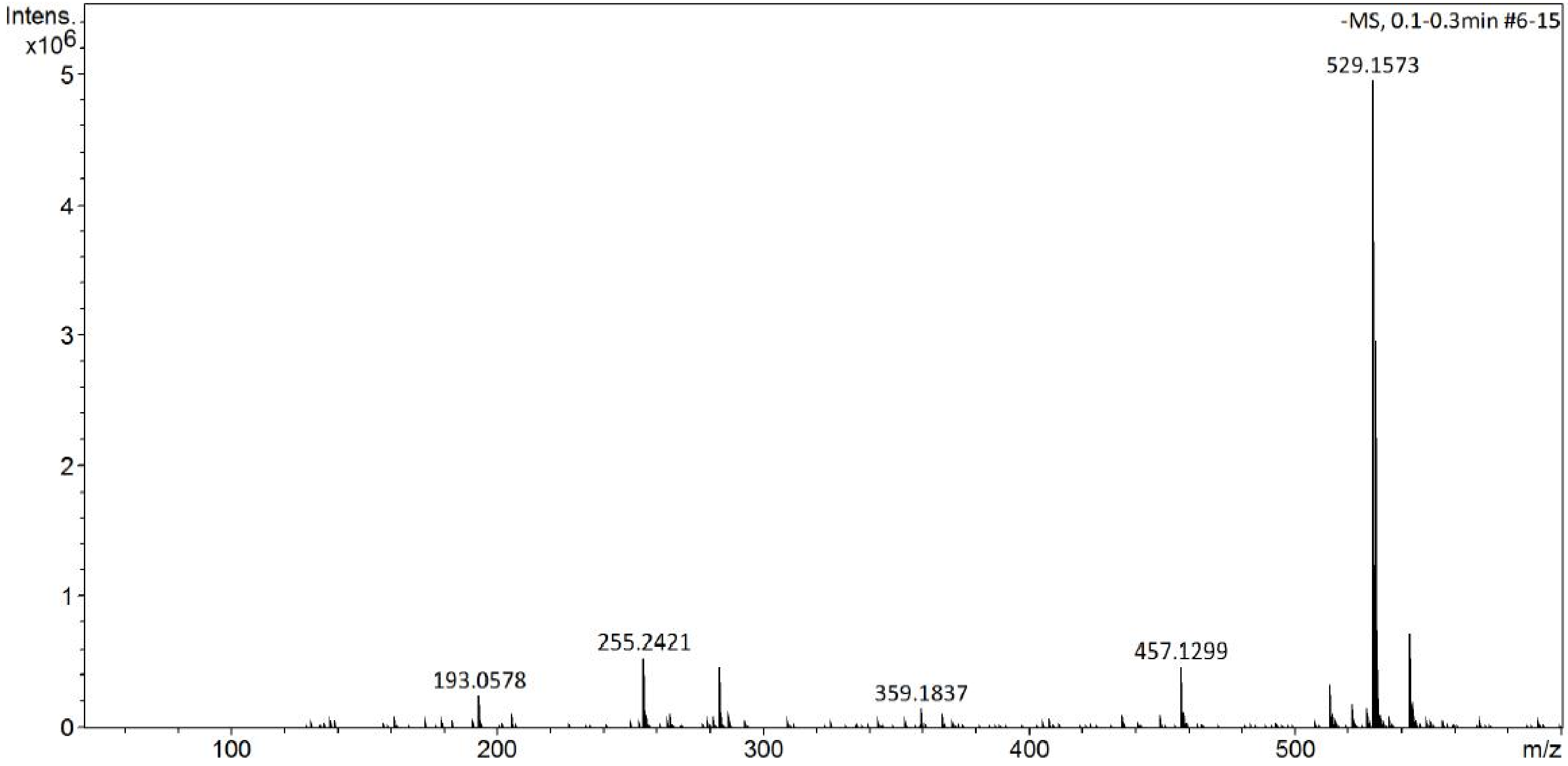

HR-ESIMS spectrum of compound **4**
